# NOD2 maybe a biomarker for the survival of kidney cancer patients

**DOI:** 10.18632/oncotarget.21547

**Published:** 2017-10-06

**Authors:** Deguo Xu, Shuisheng Zhang, Shenfeng Zhang, Hongmei Liu, Paiyun Li, Lili Yu, Heli Shang, Yong Hou, Yuan Tian

**Affiliations:** ^1^ Department of Radiation Oncology, Shandong Provincial Qianfoshan Hospital, Shandong University, Jinan, Shandong Province 250014, P.R. China; ^2^ Department of Abdominal Surgical Oncology, National Cancer Center/Cancer Hospital, Chinese Academy of Medical Sciences and Peking Union Medical College, Beijing 100021, P.R. China; ^3^ Department of Oncology, Zaozhuang Municipal Hospital of Shandong Province, Shizhong District, Zaozhuang, Shandong Province 277101, P.R. China; ^4^ Key Laboratory of Carcinogenesis and Translational Research (Ministry of Education/Beijing), Department of Aetiology, Peking University Cancer Hospital and Institute, Beijing 100142, P.R. China

**Keywords:** NOD2 gene, kidney cancer, survival, prognosis

## Abstract

**Background:**

Nucleotide-binding oligomerization domain-containing protein 2 (NOD2) may play an important role in the outcome of kidney cancer patients. To explore the relationship between NOD2 and the prognosis of kidney cancer patients, a databank-based reanalysis was conducted.

**Materials and Methods:**

Data related to kidney cancer patients at least with survival information, was obtained mainly from The Cancer Genome Atlas (TCGA). Some clinical data, not available online, was collected by personal email to the author. Then, we reanalyzed all the data in order to make a conclusion about the relationship between NOD2 gene and the prognosis of kidney cancer patients.

**Results:**

A total of 1953 samples with NOD2 information from four databanks of The Cancer Genome Atlas (TCGA) were enrolled in this study. The results of KIPAN showed the Kaplan-Meier curve for risk groups, concordance index, and *p*-value of the log-rank testing equality of survival curves ( Concordance Index = 56.57, Log−Rank Equal Curves p=0.0009006, R^2 = 0.036/0.953, Risk Groups Hazard Ratio = 1.61 (conf. int. 1.21 ~ 2.13), *p* = 0.001005) , while a box plot across risk groups, including the *p*-value testing for difference using *t*-test (or f-test for more than two groups) was shown. There was a statistical significance for the *p* value of the result (*p* < 0.01 ). The similar results could be seen in KIRC and the fourth data (including 468 samples).

**Conclusions:**

The status of NOD2 gene maybe a biomarker for the survival of kidney cancer patients.

## INTRODUCTION

The nucleotide-binding oligomerization domain 2 (NOD2)-like receptors (NLRs) belong to evolution-conserved pattern recognition receptors (PRRs) family locating in cytoplasm, closely related to responses, innate immunity and adaptive immunity [1]. Schroder K reported that they can be triggered by exogenous pathogen-associated molecular patterns (PAMPs) or endogenous damage-associated molecular patterns (DAMPs) [2]. NLRs may play a vital role on NF-κB and mitogen activated protein kinase (MAPK) signaling pathway stimulation and expression of immune response cytokines and chemokines [3]. Stimulation of NLRs can induce innate immune response, regulate adaptive immune responses, and may also participate in carcinogenesis via regulating apoptosis [4, 5].

Formerly, it was mainly considered to be a pathogenic factor for immune inflammations [6–8]. With the development of research, we found that the NLRs family, including NOD1, NOD2 and others, may also play some role in the area of cancers. Single nucleotide polymorphisms (SNPs) of NOD1 and NOD2 have been found to be associated with risk of gastric cancers (GC) and precancerous lesions in Caucasian population [9–11]. Then, the similar result was also seen in Chinese population [12]. However, the relationship between NOD2 and kidney cancer patients was poorly understood. Although some clinical trials had been taken to investigate the role of immunotherapy for kidney cancer patients, scarcely any trials were involved in NOD2 genetic status. In order to conclude the role of NOD2 among kidney cancer patients, we downloaded the published data and made further analysis with the help of some online tools.

## RESULTS

A total of 1953 samples with NOD2 information from four databanks of TCGA were enrolled in this study. Of 1953 subjects with survival status, 468 cases were along with data about clinical grade and stage. The overall characteristic of them can be seen in Table 1. The primary data of them were listed in supplementary material. We performed all analysis in SurvExpress using the maximum row average for NOD2 with multiple probe sets, two risk groups by prognostic index median, and Cox fitting.

**Table 1:** The overall characteristics of four dataset

NO	Database	Number of samples	Clinical informationof the data	Source of the data
1	KIPAN - TCGA Kidney PAN cancer TCGA June 2016	792	Survival	TCGA
2	KIRC - TCGA - Kidney renal clear cell carcinoma	415	Survival	TCGA
3	KIRP - TCGA Kidney renal papillary cell carcinoma June 2016	278	Survival	TCGA
4	kidney renal clear cell carcinoma TCGA	468	Survival, Grade, Stage	TCGA

**Figure 1:**
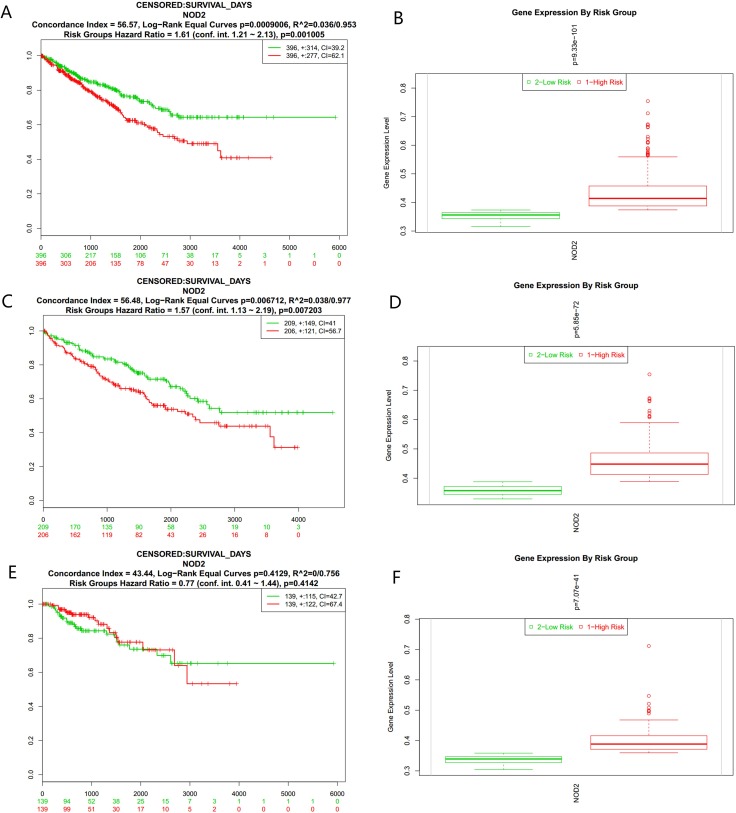
(**A**) Kaplan-Meier curve for risk groups, concordance index (CI), and *p*-value of the log-rank testing equality of survival curves in KIPAN. Red and Green curves denote High- and Low-risk groups respectively. The ordinal (Y-axis) indicates the percentage of survival, the abscissa (X-axis) represents survival days, and the number of survivors at the corresponding time. Censoring samples are shown as “+” marks. The number of individuals, the number of censored, and the CI of each risk group are shown in the top-right insets. (**B**) Box plot across risk groups, including the *p*-value testing for difference using *t*-test (or f-test for more than two groups) in KIPAN. The ordinate (Y-axis) means the expression percentage of the gene. the abscissa (X-axis) represents different risk groups. (**C**) Kaplan-Meier curve for risk groups, concordance index (CI), and *p*-value of the log-rank testing equality of survival curves in KIRC. Red and Green curves denote High- and Low-risk groups respectively. The ordinal (Y-axis) indicates the percentage of survival, the abscissa (X-axis) represents survival days, and the number of survivors at the corresponding time. Censoring samples are shown as “+” marks. The number of individuals, the number of censored, and the CI of each risk group are shown in the top-right insets. (**D**) Box plot across risk groups, including the *p*-value testing for difference using *t*-test (or f-test for more than two groups) in KIRC. The ordinate (Y-axis) means the expression percentage of the gene. the abscissa (X-axis) represents different risk groups. (**E**) Kaplan-Meier curve for risk groups, concordance index (CI) , and *p*-value of the log-rank testing equality of survival curves in KIRP. Red and Green curves denote High- and Low-risk groups respectively. The ordinal (Y-axis) indicates the percentage of survival, the abscissa (X-axis) represents survival days, and the number of survivors at the corresponding time. Censoring samples are shown as “+” marks. The number of individuals, the number of censored, and the CI of each risk group are shown in the top-right insets. (**F**) Box plot across risk groups, including the *p*-value testing for difference using *t*-test (or f-test for more than two groups) in KIRP. The ordinate (Y-axis) means the expression percentage of the gene. the abscissa (X-axis) represents different risk groups.

We analyzed the data only with survival information first. Among all the results, green color represents low-risk groups, and red color means high-risk groups. Figure 1A showed the Kaplan-Meier curve for risk groups, concordance index (CI), and *p*-value of the log-rank testing equality of survival curves (CI = 56.57, Log−Rank Equal Curves *p* = 0.0009006, R^2 = 0.036/0.953, Risk Groups Hazard Ratio = 1.61 (conf. int. 1.21 ~ 2.13), *p* = 0.001005) of KIPAN, as recommended by Bovelstad HM [13], while a box plot across risk groups, including the *p*-value testing for difference using *t*-test (or f-test for more than two groups) was shown in Figure 1B. There was a statistical significance for the *P* value of the result (*P* < 0.01 ). From the above analysis, we found that the expression level of NOD2 gene might be a bad signal for the prognosis of kidney cancer patients. Then,we analyzed another two data from TCGA, named KIRC and KIRP, but the similar results were only observed in KIRC (Figure 1C and Figure 1D). As is shown in Figure 1E and Figure 1F (CI = 43.44, Log−Rank Equal Curves *p* = 0.4129, R^2=0/0.756, Risk Groups Hazard Ratio = 0.77 (conf. int. 0.41 ~ 1.44), *p* = 0.4142), the *p* value of KIRP is of no statistical significance.

**Figure 2:**
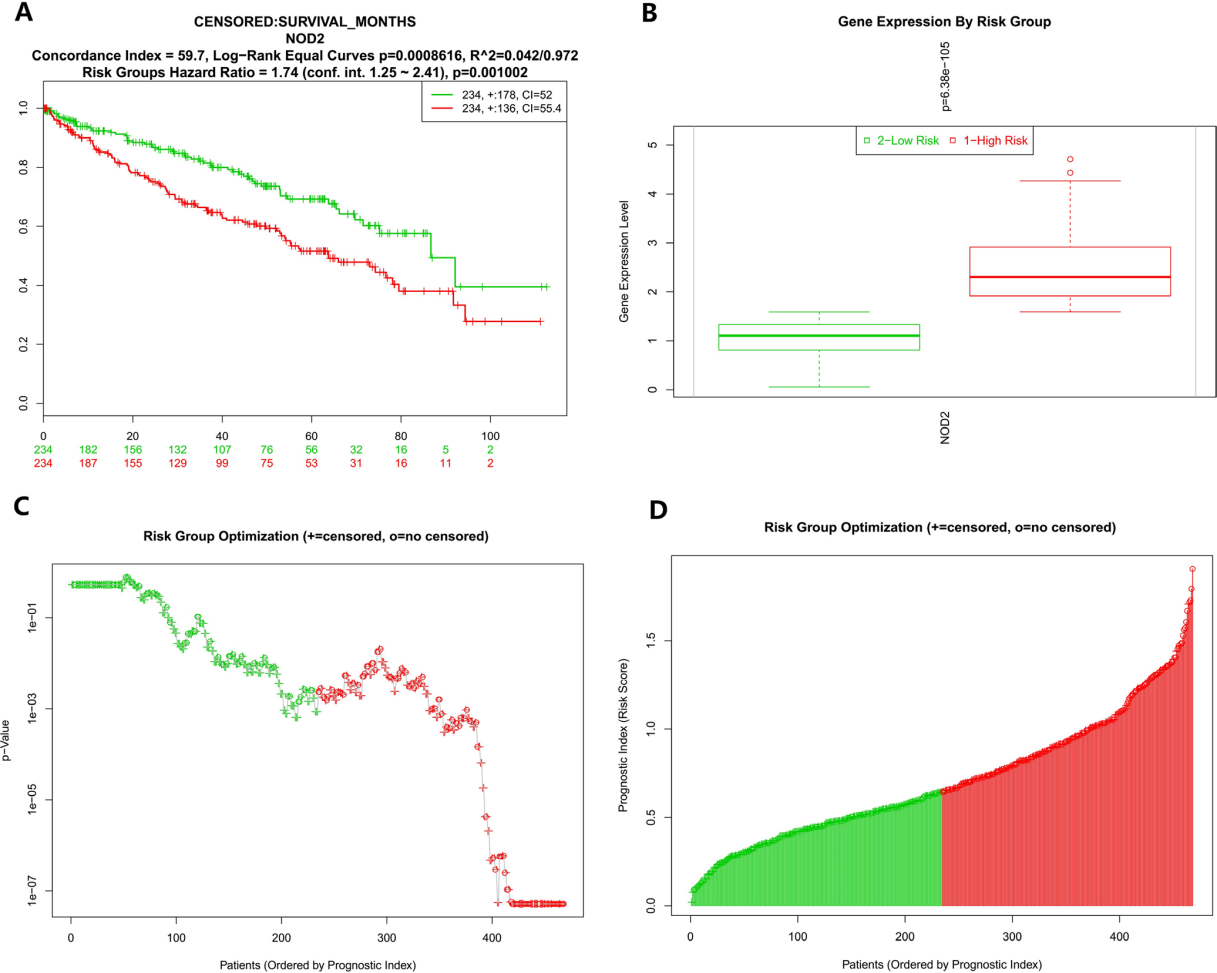
(**A**) Kaplan-Meier curve for risk groups, concordance index (CI), and *p*-value of the log-rank testing equality of survival curves. Red and Green curves denote High- and Low-risk groups respectively. The ordinal (Y-axis) indicates the percentage of survival, the abscissa (X-axis) represents survival days, and the number of survivors at the corresponding time. Censoring samples are shown as “+” marks. The number of individuals, the number of censored, and the CI of each risk group are shown in the top-right insets. (**B**) Box plot across risk groups, including the *p*-value testing for difference using *t*-test (or f-test for more than two groups). The ordinate (Y-axis) indicates the expression percentage of the gene. the abscissa (X-axis) represents different risk groups. (**C**) The process of risk group optimization. The ordinate (Y-axis) indicates *p* value. the abscissa (X-axis) represents patients ordered by prognostic index. “+” = censored. “o”= no censored. (**D**) The process of risk group optimization. The ordinate (Y-axis) indicates risk score of prognostic index, the abscissa (X-axis) represents patients ordered by prognostic index. “+” = censored. “o”= no censored.

**Figure 3:**
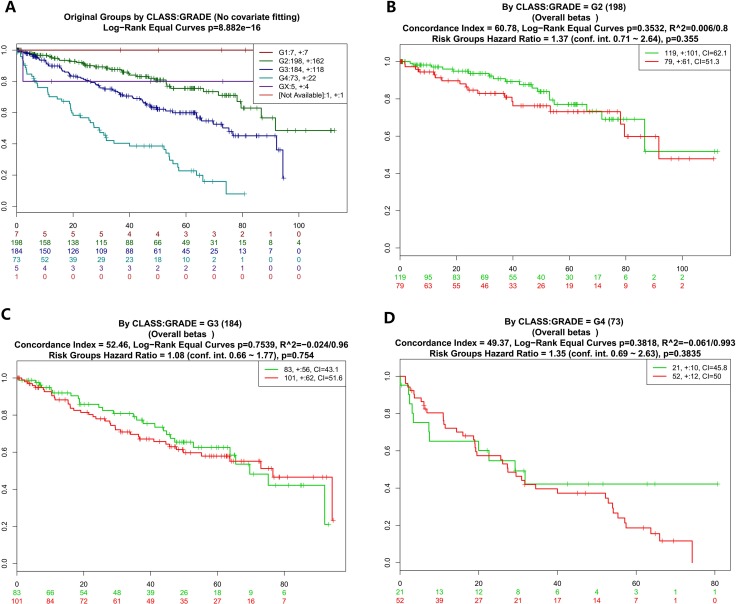
Kaplan-Meier curves and performance of stratification analysis in the kidney cancer data according to tumor grades Red and Green curves denote High- and Low-risk groups respectively. The ordinal (Y-axis) indicates the percentage of survival, the abscissa (X-axis) represents survival days, and the number of survivors at the corresponding time. Censoring samples are shown as “+” marks. The number of individuals, the number of censored, and the CI of each risk group are shown in the top-right insets. (**A**) Kaplan-Meier curves and performance of stratification analysis for original groups by class:grade (No covariate fitting). (**B**) Kaplan-Meier curves and performance of stratification analysis by class:grade = G2. (**C**) Kaplan-Meier curves and performance of stratification analysis by class:grade = G3. (**D**) Kaplan-Meier curves and performance of stratification analysis by class:grade = G4.

**Figure 4:**
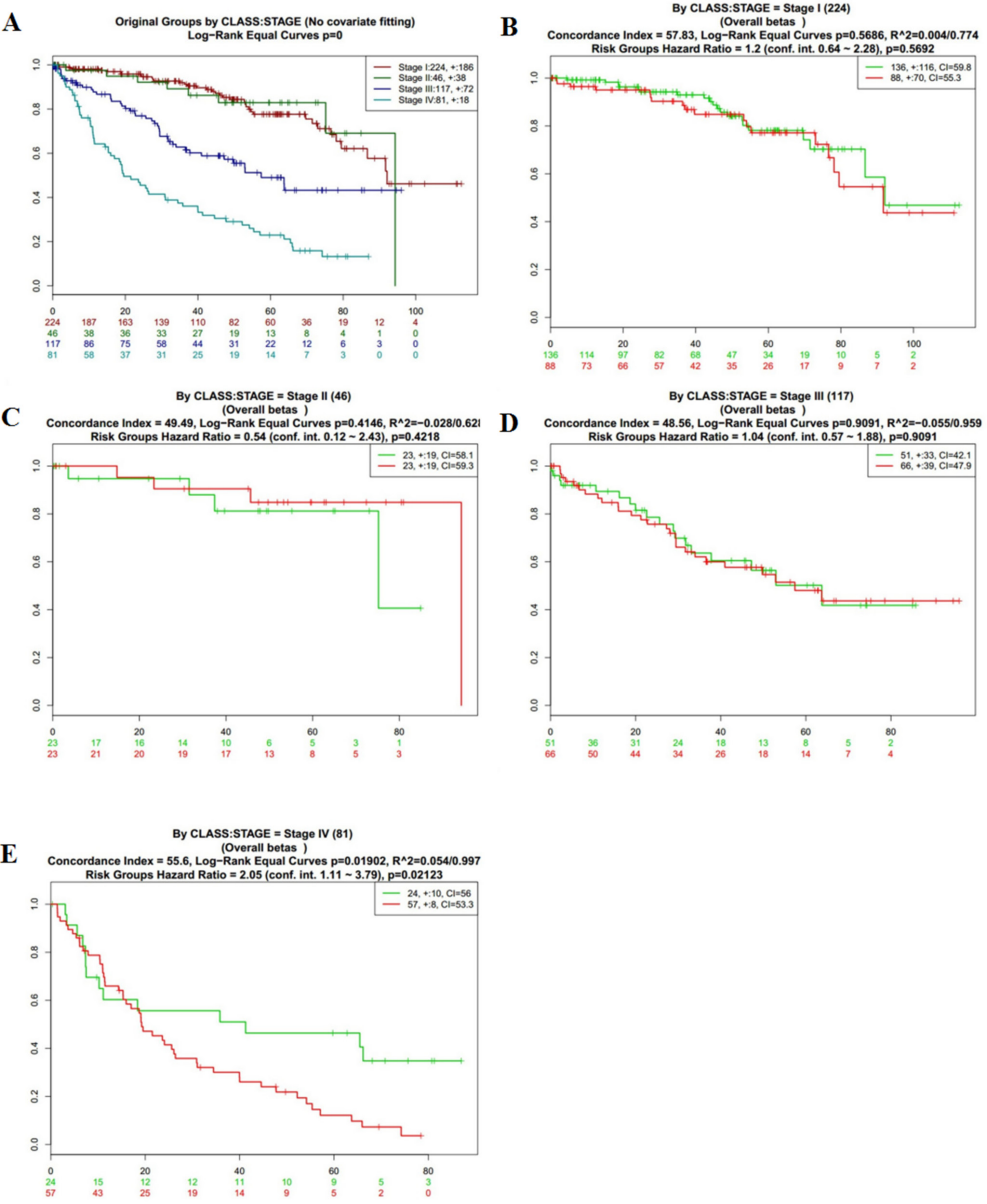
Kaplan-Meier curves and performance of stratification analysis in the kidney cancer data according to tumor related death Red and Green curves denote High- and Low-risk groups respectively. The ordinal (Y-axis) indicates the percentage of survival, the abscissa (X-axis) represents survival days, and the number of survivors at the corresponding time. Censoring samples are shown as “+” marks. The number of individuals, the number of censored, and the CI of each risk group are shown in the top-right insets. (**A**) Kaplan-Meier curves and performance of stratification analysis for original groups by class:stage (No covariate fitting). (**B**) Kaplan-Meier curves and performance of stratification analysis by stage I. (**C**) Kaplan-Meier curves and performance of stratification analysis by stage II. (**D**) Kaplan-Meier curves and performance of stratification analysis by stage III. (**E**) Kaplan-Meier curves and performance of stratification analysis by stage IV.

**Figure 5:**
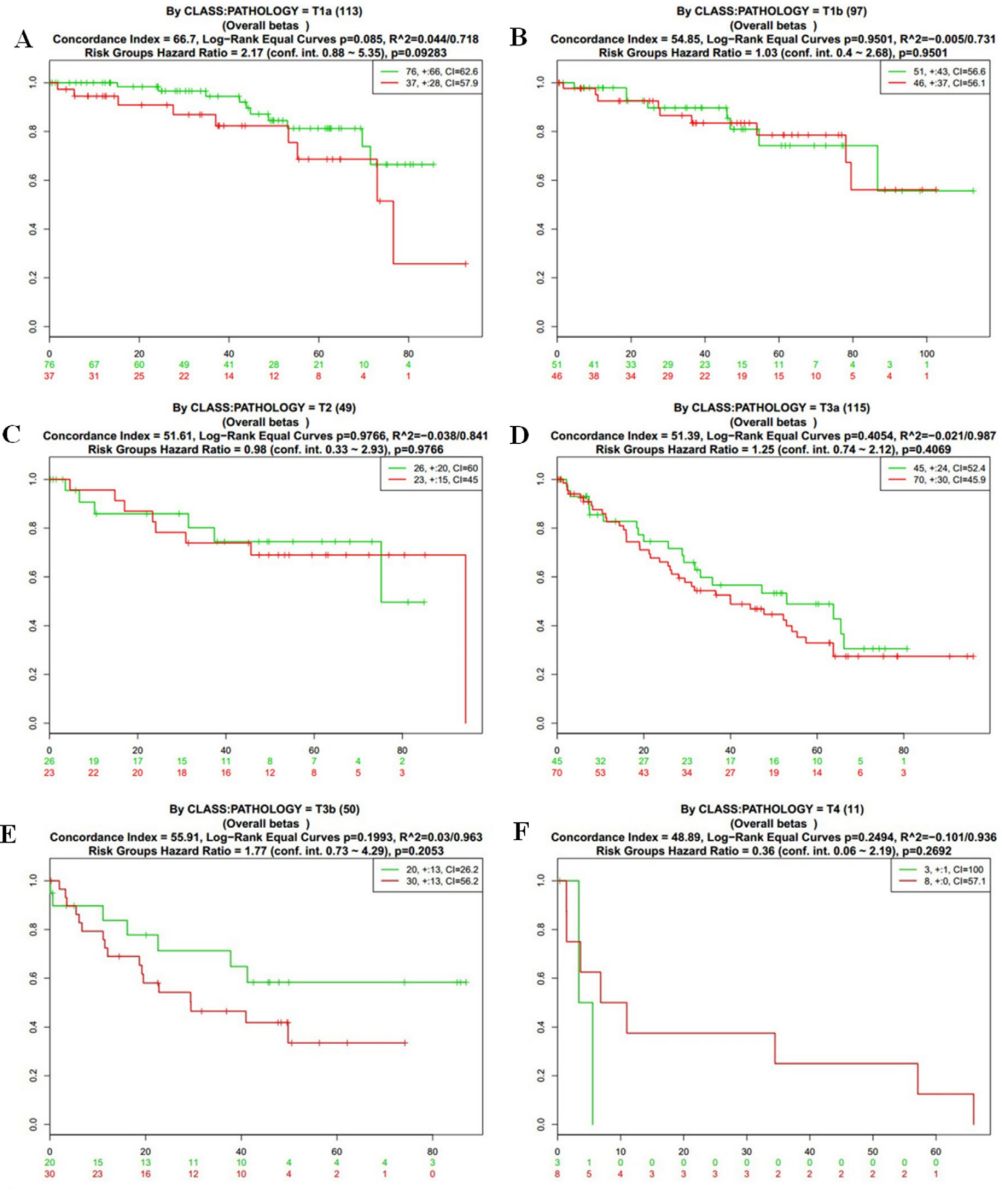
Kaplan-Meier curves and performance of stratification analysis in the kidney cancer data according to tumor stage Red and Green curves denote High- and Low-risk groups respectively. The ordinal (Y-axis) indicates the percentage of survival, the abscissa (X-axis) represents survival days, and the number of survivors at the corresponding time. Censoring samples are shown as “+” marks. The number of individuals, the number of censored, and the CI of each risk group are shown in the top-right insets. (**A**) Kaplan-Meier curves and performance of stratification analysis by pathology = T1a. (**B**) Kaplan-Meier curves and performance of stratification analysis by pathology = T1b. (**C**) Kaplan-Meier curves and performance of stratification analysis by pathology = T2. (**D**) Kaplan-Meier curves and performance of stratification analysis by pathology = T3a. (**E**) Kaplan-Meier curves and performance of stratification analysis by pathology = T3b. (**F**) Kaplan-Meier curves and performance of stratification analysis by pathology = T4.

**Figure 6:**
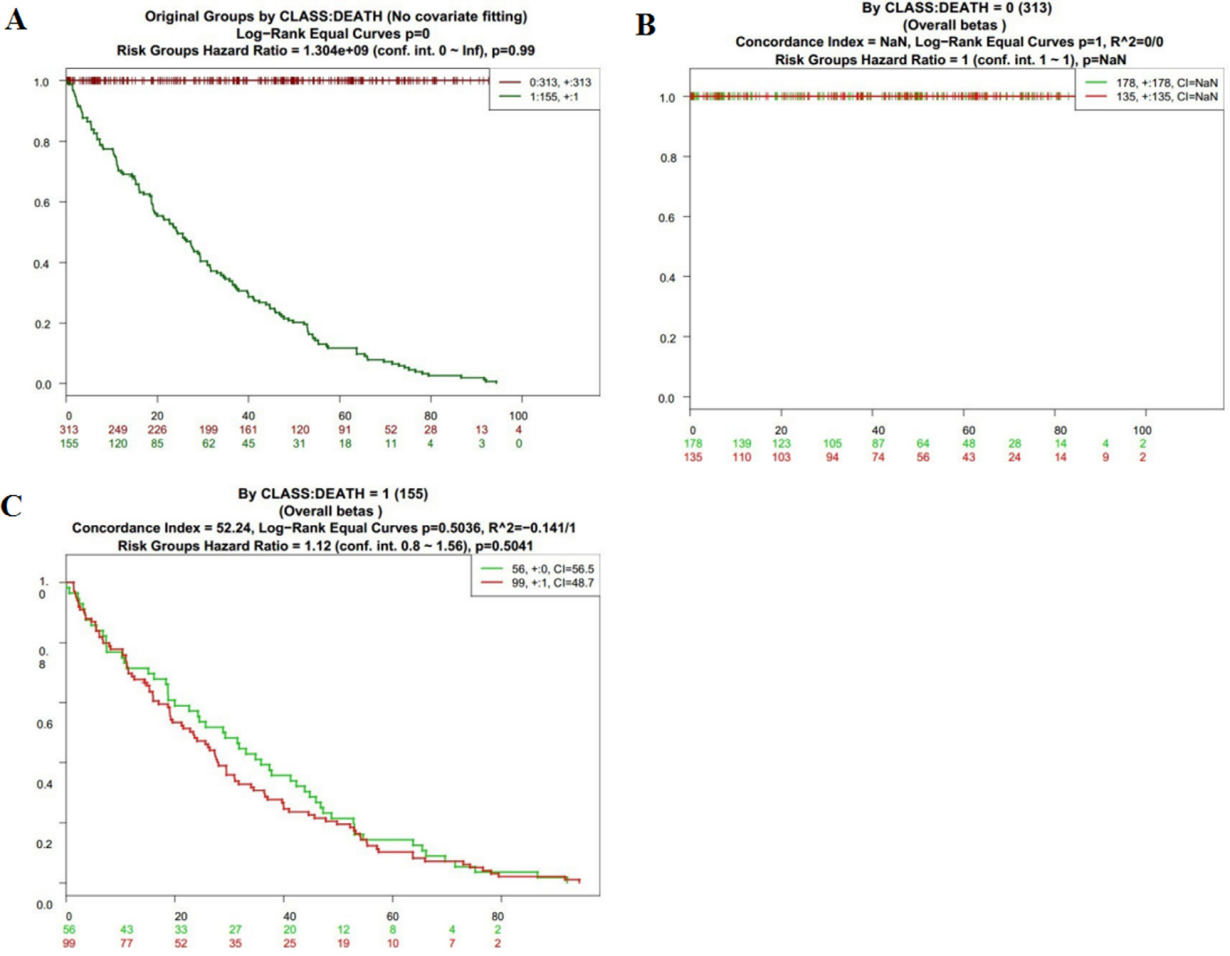
Kaplan-Meier curves and performance of stratification analysis in the kidney cancer data according to tumor related death Red and Green curves denote High- and Low-risk groups respectively. The ordinal (Y-axis) indicates the percentage of survival, the abscissa (X-axis) represents survival days, and the number of survivors at the corresponding time. Censoring samples are shown as “+” marks. The number of individuals, the number of censored, and the CI of each risk group are shown in the top-right insets. (**A**) Kaplan-Meier curves and performance of stratification analysis for original groups by class:death (No covariate fitting). (**B**) Kaplan-Meier curves and performance of stratification analysis by class: death = 0. (**C**) Kaplan-Meier curves and performance of stratification analysis by class: death = 1.

To further analyze the data about NOD2 gene expressive level, we tested the NOD2 in the fourth database from TCGA that contains 468 samples with survival, grade and stage data using the SurvExpress stratification functionality. The overall results of the fourth data were summarized in Figure 2A and 2B (CI = 59.7, Log−Rank Equal Curves p=0.0008616, R^2=0.042/0.972, Risk Groups Hazard Ratio = 1.74 (conf. int. 1.25 ~ 2.41), *p* = 0.001002), which *p* value was of notable significance. Then,the stratification analysis of 468 samples was made according to grade, stage, pathology, and death of the tumor data.. The Log−Rank Equal Curves were obviously separated from each other in Figure 3A and Figure 4A, when all the patients were grouped by tumor grade and stage. However, when every subgroup was divided into two risk groups in Figure 3B–3E and Figure 4B–4D, the results of stratification analysis for every stage patients were ambiguous except for Figure 4E. No statistical significance was observed. Similar indefinite results of risk subgroup stratification analysis, according to pathology and death of the tumor data,could also be seen. They were gathered in Figure 5 and Figure 6. The details of the stratification analysis results were displayed in Table 2.

**Table 2:** The stratification reanalysis results of 468 samples

Stratification Class	The Detail of Stratification	Concordance Index	Log−Rank Equal Curves (p)	R^2	Risk Groups Hazard Ratio	*p*
**Grade (Figure 3)**	G2 (198)	60.78	0.3532	0.006/0.8	1.37 (conf. int. 0.71 ~ 2.64)	0.355
	G3 (184)	52.46	0.7539	−0.024/0.96	1.08 (conf. int. 0.66 ~ 1.77)	0.754
	G4 (73)	49.37	0.3818	−0.061/0.993	1.35 (conf. int. 0.69 ~ 2.63)	0.3835
**Stage (Figure 4)**	Stage I (224)	57.83	0.5686	0.005/0.774	1.2 (conf. int. 0.64 ~ 2.28)	0.5692
	Stage II (46)	49.49	0.4146	−0.031/0.628	0.54 (conf. int. 0.12 ~ 2.43)	0.4218
	Stage III (117)	48.56	0.9091	−0.059/0.959	1.04 (conf. int. 0.57 ~ 1.88)	0.9091
	Stage IV (81)	55.6	0.01902	0.063/0.997	2.05 (conf. int. 1.11 ~ 3.79)	0.02123
**Pathology (Figure 5)**	T1 (18)	64.52	0.6802	−0.005/0.649	1.47 (conf. int. 0.24 ~ 9.11)	0.6818
	T1a (113)	66.7	0.085	0.045/0.718	2.17 (conf. int. 0.88 ~ 5.35)	0.09283
	T1b (97)	54.97	0.9501	−0.007/0.731	1.03 (conf. int. 0.4 ~ 2.68)	0.9501
	T2 (49)	51.61	0.9766	−0.038/0.841	0.98 (conf. int. 0.33 ~ 2.93)	0.9766
	T3a (115)	51.39	0.4054	−0.03/0.987	1.25 (conf. int. 0.74 ~ 2.12)	0.4069
	T3b (50)	55.91	0.1993	0.031/0.963	1.77 (conf. int. 0.73 ~ 4.29)	0.2053
	T4 (11)	48.89	0.2494	−0.114/0.936	0.36 (conf. int. 0.06 ~ 2.19)	0.2692
**Death (Figure 6)**	0 (313)	NaN	1	0/0	1 (conf. int. 1 ~ 1)	NaN
	1 (155)	52.25	0.5036	−0.127/1	1.12 (conf. int. 0.8 ~ 1.56)	0.5041

## DISCUSSION

There are nearly 270,000 patients suffering kidney cancer, which leads to over 115,000 deaths each year [14]. As known, kidney cancer is comprised of many different histological and genetical types of cancer, and the histological type of cancer is associated with clinical course and responses to treatment [15, 16]. If the kidney tumor is small, it can be surgically removed and makes patients acquire an expectation of a 5- to 10-years survival. However, if a patient presents with metastasized kidney cancer, nearly 80% will die of this disease within 2 years [17]. Multiple genes linked with kidney cancer, including the VHL, MET, FLCN, fumarate hydratase, succinate dehydrogenase, TSC1, TSC2, and TFE3, were identified by genomic studies and have significantly altered the ways in which patients with kidney cancer are managed.

Though, seven FDA-supported agents, targeting the VHL pathway, had been admitted for the therapy of patients with advanced kidney cancer, further genomic studies, such as whole genome sequencing, gene expression patterns, and so on, will still be needed to get a complete understanding of the genetic basic mechanism of kidney cancer and the kidney cancer gene pathways and, most importantly, to provide the foundation for the development of effective forms of therapy for patients with the disease [18]. More and more new cancer-related genes are required to be investigated in order to provide useful guidance for cancer treatments.

The NOD2-like receptors (NLRs) belong to evolution-conserved pattern recognition receptors (PRRs) family locating in cytoplasm, closely related to responses, innate immunity and adaptive immunity [1]. NLRs are expressed in various cell types, including macrophage, neutrophils, epithelial and endothelial cells, dendritic cells, as well as in malignant tumors [19]. Genetic variations in NOD1 and NOD2 are associated with increased susceptibility to Crohn's disease [20]. A.Marijke Keestra-Gounder found that NOD1 and NOD2, two members of the NLR family of PRRs, are important mediators of ER stress-induced inflammation. The association of NOD1 and NOD2 with pro-inflammatory responses induced by the IRE1-α/TRAF2 signaling pathway provides a novel link between innate immunity and ER stress-induced inflammation [21].

With the development of whole genome sequencing, plenty of data about NOD2 gene in cancers can be acquired online, such as TCGA, Gene Expression Omnibus (GEO) and so on. Previously, most of us paid our attention to the relationship between NOD2 and common cancers except for kidney cancers. Some databases contained some information about the NOD2 gene, but we ignored its existence. Therefore, we tried to reanalyze the data online to make sure the role of NOD2 in kidney cancer patients.

The data used for reanalysis represented a wide variety of sample sizes. To achieve the results that would be representative of the greatest number of study parameters possible, all reanalysis included as much data information as possible. The analysis results of four TCGA data, including the Kaplan-Meier curve for risk groups, concordance index (CI), and *p*-value of the log-rank testing equality of survival curves, were shown in corresponding Figures 1–6.

The NOD2 expressive level seemed to be an adverse event for the survival of kidney cancer patients in KIPAN and KIRC database, which is obvious in Figure 1A and Figure 1C ,while a box plot across risk groups in Figure 1B and Figure 1D also showed the similar approval testimony. As the figure showed, the survival Kaplan-Meier curves, displayed in green and red colors, were separated distinctly from each other and *p*-value of the log-rank test was of statistically significant. The concordance index (CI) is one of the most commonly used performance measures of survival models. It can be interpreted as the fraction of all pairs of subjects whose predicted survival times are correctly ordered among all subjects that can actually be ordered. In other words, it is the probability of concordance between the predicted and the observed survival. The concordance index (CI = 56.57 and 56.48) of KIPAN and KIRC suggest that the the fraction of all pairs of samples whose predicted survival times are correctly ordered.

The reanalysis results of KIRP, shown in Figure 1E and 1F, were of no statistical significance (CI = 43.44, Log−Rank Equal Curves *p* = 0.4129, R^2 = 0/0.756,Risk Groups Hazard Ratio = 0.77 (conf. int. 0.41 ~ 1.44), *p* = 0.4142). After checking up the primary data of KIRP, which is listed in the supplementary material, we could not find out the definite reason for it. In order to collect more information about the NOD2 gene in kidney cancers and find out the reason for the result of KIRP, we use SurvExpress to put stratification of 468 samples into practice according to death, grade, stage, and pathology. The detail of the reanalysis that was similar to KIPAN and KIRC ,was arranged in the form of graphs in Figure 2. Our reanalysis of data stated clearly that the NOD2 gene may have something to do with the survival of kidney cancers. The relevance was also endorsed by stratification analysis in Figure 3A and Fgiure 4A respectively according to tumor grade and stage. Although all survival curves showed a separate trend in the deeper stratification analysis of 468 samples, the *p* value was not statistically significant. I deduced that the emergence of such results may be related to limited samples assigned to different groups.The deeper analysis results of the fourth database also showed the inclination that the NOD2 expressive level was an unfavorable signal for the survival of kidney cancer patients.

A lot of researches revealed that the NOD2 gene may be relevant to caners [21–24], especially in gastric cancers, but it is the first time that the relationship between NOD2 gene and the prognosis of kidney cancers was revealed by a reanalysis of sequencing data. We clearly stated the close relationship between the NOD2 gene and tumor stage on the survival of kidney cancer patients by deeper stratification analysis. In the future, with the increase in kidney cancer patients data and the development of science, more and more unknown relevance of NOD2 gene may be elucidated. In the present analysis, no enough evidence was promulgated to authenticate the existing association between NOD2 and kidney cancers. More sequencing data and studies on the mechanism of cancer occurrence and clinical trials about NOD2 gene, such as inflammation and immune disease area [25, 26], are needed to further reveal the specific mechanism of action in kidney cancer areas.

## MATERIALS AND METHODS

### Data collection

We got all the data mainly from TCGA using keywords related to NOD2, kidney cancer, survival, and gene expression technologies. From TCGA, all data were obtained at the gene level (level 3). RNA-Seq counts data were log2 transformed. Then, we analyzed all the data with the help of online tools as ITTACA, KMPlot, Recurrence Online, bc-GeneExMiner, GOBO, PrognoScan and SurvExpress. We found that SurvExpress was most convenient online tool for this analysis, which is available in (http://bioinformatica.mty.itesm.mx/SurvExpress). It includes a tutorial that describes the analysis options, plots, tables, key concepts related to survival analysis, and representative methods to identify biomarker from gene expression data. The Characteristics of all the data was shown in Table 1.

### Prognostic index estimation

During the process of this analysis, we mainly paid our attention to the prognostic index (PI), also named as the risk score sometimes, usually taken to generate risk groups and the linear component of the Cox model [27]. PI = β1×1+ β2×2+...+βpxp. Each β_I_ can be considered as a risk coefficient. SurvExpress that is better than others for more procedures was adopted to estimate the β coefficients. The first one is the classical Cox model ,all genes are included in a unique model,as is performed in R (http://cran.r-project.org) using the survival package. Second, we can make sure a weight for each gene but using the values from the Cox fitting.

The concordance index (CI) is one of the most commonly used performance measures of survival models. It can be interpreted as the fraction of all pairs of subjects whose predicted survival times are correctly ordered among all subjects that can actually be ordered [28]. In other words, it is the probability of concordance between the predicted and the observed survival. The CI, which quantifies the quality of rankings, is the standard performance measure for model assessment in survival analysis. Survival rate was plotted using Kaplan−Meier method and analyzed using Log-rank test. The frequencies of categorical variables were compared using Pearson χ^2^ or Fisher's exact test, when appropriate. A value of *P* < 0.01 was considered to be significant.

### Risk estimation

We take the first method to generate the risk groups splitting the ordered PI (higher values corresponding to higher risk) and then leave equal number of samples in each group to mark the number of risk groups. If there are two risk groups, we will split the PI by the median. The second method producing risk groups takes an optimization algorithm for the ordered PI. In a word, for two groups, the log-rank test will be implemented along all values of the arranged PI. Then,the minimum *p*-value is chosen as the split point for the algorithm. This procedure is generalized for more than two groups repeatedly optimizing one risk group at the time until no changes are found. All the process of risk estimation can be easily finished by the online analysis tools [29]. In this survival analysis, the hazard ratio (HR) is the ratio of the hazard rates corresponding to the conditions described by two levels of risk groups.

## SUPPLEMENTARY MATERIALS FIGURES AND TABLES











## Abbreviations

NOD2: nucleotide-binding oligomerization domain-containing protein 2
NLRs: nucleotide -binding oligomerization domain 2 -like receptors
PRRs: pattern recognition receptors
CI: concordance index
PI: prognostic index
TCGA: The Cancer Genome Atlas
DAMPs: damage-associated molecular patterns
HR: hazard ratio
GC: gastric cancer
MAPK: mitogen activated protein kinase
GEO: Gene Expression Omnibus
