# Supplementary material for: NOD2 maybe a biomarker for the survival of kidney cancer patients
Source: Oncotarget. 2017 Oct 6;8(60):101489–99. doi: 10.18632/oncotarget.21547 (PMC5731890; doi:10.18632/oncotarget.21547)
Supplement: Supplementary file 1 [file oncotarget-08-101489-s001.pdf]

NOD2 maybe a biomarker for the survival of kidney cancer patients

SUPPLEMENTARY MATERIALS

Supplementary Material 1: Primary survival data of KIPAN. See Supplementary_Material_1

Supplementary Material 2.Primary survival data of KIRC. See Supplementary_Material_2

Supplementary Material 3: Primary survival data of KIRP. See Supplementary_Material_3

Supplementary Material 4: Primary data of 468 samples. See Supplementary_Material_4
