# Supplementary material for: NOD2 maybe a biomarker for the survival of kidney cancer patients
Source: Oncotarget. 2017 Oct 6;8(60):101489–99. doi: 10.18632/oncotarget.21547 (PMC5731890; doi:10.18632/oncotarget.21547)
Supplement: Supplementary file 3 [file oncotarget-08-101489-s003.doc]

**#CENSORED:SURVIVAL_DAYS**

16+ 1191 1493+ 1491+ 1130+ 1508+ 1478+ 1106+ 1137+ 137 1610 1385+ 2688+ 1307+ 1559+ 319+ 873+ 2270+ 2274+ 735+ 1621+ 1696 630+ 567+ 574+ 861+ 1993+ 617+ 3343+ 3728+ 3328+ 2241 2087+ 3331+ 1471+ 2392+ 1779+ 2868+ 874+ 683 1143+ 1168+ 2508+ 2217+ 43+ 43 139 77 106 578 1980 182 65 600 1755+ 1337 202 99 1778+ 478 1417 18 168 1588 1371 1019 510 183 1111 454 1657 885 307 2386 1238 1378 834 1639 204 1724 320 313 1986 1200 793 883 69 1121 2145 637 1317 342 362 770 563 459 333 245 68 665+ 485 1913 2764 2752 1598 927 1782+ 587 2009+ 1175+ 1604+ 1274+ 1552+ 1169+ 1485+ 1411+ 1733+ 1290+ 3431+ 3944+ 4074+ 480 2150+ 2609+ 2630+ 3631+ 3841+ 2461+ 2172+ 2246+ 4537+ 3205+ 3744+ 3974+ 2430+ 3989+ 2782+ 3834+ 1092+ 51 972+ 648+ 963+ 755+ 656+ 365+ 709 511+ 1520+ 1299+ 762+ 1380+ 777+ 788+ 1525+ 1218+ 722+ 822+ 26+ 737+ 1525+ 505+ 194+ 1476+ 16+ 1046+ 435+ 3377+ 2601 2881+ 2746+ 3074+ 2839+ 992 3037+ 13+ 2718+ 701 2343 1893+ 1879+ 1955+ 1625 845 1888+ 2454 2 2859+ 562 1589 2256 1912 1516+ 1493 1367+ 372+ 970+ 344 1034 953 374+ 2208+ 2372+ 182+ 1343 1270 2184+ 1462+ 400+ 329 162 1885+ 1843+ 1731+ 2080+ 354+ 480 1489+ 1111 1107+ 334 1133 1124+ 204+ 1459+ 211+ 2172+ 1935+ 1785+ 1834+ 1862+ 205+ 1746+ 433+ 1487+ 1502+ 1384+ 211 1433+ 1632+ 454+ 1097 1014+ 1413+ 952 785+ 1124+ 828 118+ 1413+ 501+ 177+ 1371+ 932+ 1266+ 563+ 1177+ 1126+ 840+ 1140+ 1071+ 1092 878 1463 193+ 2412+ 62 2257+ 932+ 1590 293+ 1912 2263+ 1165+ 1291+ 1133+ 1132+ 693+ 406+ 822 1011+ 967+ 714+ 408+ 749+ 1018+ 603+ 1355+ 1063+ 951+ 29+ 3498+ 1416+ 1924+ 2227 431 3229+ 3480+ 1661 1793+ 336 646 1498+ 2423+ 1435+ 2259+ 2283+ 3554 1955+ 2186+ 2014+ 1883+ 1759+ 3451+ 1952+ 932 1567 1946+ 3519+ 819 1521+ 750+ 841 1200 3341+ 1528+ 1450+ 1520+ 1560+ 3302+ 1496+ 1499+ 1531+ 1657+ 1373+ 93 139 572 3987+ 2190 3936+ 4067+ 782 574 679 768 552 3736+ 1889+ 2231+ 2038+ 3615 1625 2299 1906+ 3639+ 224 1964 2799+ 2489+ 2609+ 2017+ 2378+ 2271+ 41 2552+ 3146+ 774+ 1404 18+ 1432 2105 1787+ 446 3271+ 1928+ 166 3267+ 1929+ 1789+ 2419 722 561 2422+ 2754+ 2789+ 1683+ 2873+ 330 311 662+ 2564 685+ 73 59 946 386+ 2439+ 2067+ 1997+ 373+ 445 693+ 1905+ 1329+ 1398+ 370+ 355+ 2016+ 1729+ 727 551+ 119+ 206+ 127+

**SYMBOL:NOD2 Gene**

DATA:TCGA.A3.3308 DATA:TCGA.A3.3311 DATA:TCGA.A3.3316 DATA:TCGA.A3.3317 DATA:TCGA.A3.3319 DATA:TCGA.A3.3320 DATA:TCGA.A3.3322 DATA:TCGA.A3.3323 DATA:TCGA.A3.3326 DATA:TCGA.A3.3346 DATA:TCGA.A3.3347 DATA:TCGA.A3.3349 DATA:TCGA.A3.3357 DATA:TCGA.A3.3358 DATA:TCGA.A3.3362 DATA:TCGA.A3.3363 DATA:TCGA.A3.3365 DATA:TCGA.A3.3367 DATA:TCGA.A3.3370 DATA:TCGA.A3.3372 DATA:TCGA.A3.3373 DATA:TCGA.A3.3376 DATA:TCGA.A3.3378 DATA:TCGA.A3.3380 DATA:TCGA.A3.3382 DATA:TCGA.A3.3383 DATA:TCGA.A3.3385 DATA:TCGA.A3.3387 DATA:TCGA.AK.3425 DATA:TCGA.AK.3428 DATA:TCGA.AK.3429 DATA:TCGA.AK.3431 DATA:TCGA.AK.3434 DATA:TCGA.AK.3436 DATA:TCGA.AK.3444 DATA:TCGA.AK.3445 DATA:TCGA.AK.3450 DATA:TCGA.AK.3451 DATA:TCGA.AK.3454 DATA:TCGA.AK.3455 DATA:TCGA.AK.3456 DATA:TCGA.AK.3458 DATA:TCGA.AK.3460 DATA:TCGA.AK.3461 DATA:TCGA.AS.3778 DATA:TCGA.B0.4690 DATA:TCGA.B0.4691 DATA:TCGA.B0.4693 DATA:TCGA.B0.4694 DATA:TCGA.B0.4697 DATA:TCGA.B0.4700 DATA:TCGA.B0.4703 DATA:TCGA.B0.4706 DATA:TCGA.B0.4707 DATA:TCGA.B0.4710 DATA:TCGA.B0.4712 DATA:TCGA.B0.4713 DATA:TCGA.B0.4714 DATA:TCGA.B0.4718 DATA:TCGA.B0.4810 DATA:TCGA.B0.4811 DATA:TCGA.B0.4813 DATA:TCGA.B0.4814 DATA:TCGA.B0.4815 DATA:TCGA.B0.4816 DATA:TCGA.B0.4817 DATA:TCGA.B0.4818 DATA:TCGA.B0.4819 DATA:TCGA.B0.4822 DATA:TCGA.B0.4823 DATA:TCGA.B0.4824 DATA:TCGA.B0.4827 DATA:TCGA.B0.4828 DATA:TCGA.B0.4833 DATA:TCGA.B0.4836 DATA:TCGA.B0.4837 DATA:TCGA.B0.4838 DATA:TCGA.B0.4839 DATA:TCGA.B0.4841 DATA:TCGA.B0.4842 DATA:TCGA.B0.4843 DATA:TCGA.B0.4844 DATA:TCGA.B0.4845 DATA:TCGA.B0.4846 DATA:TCGA.B0.4847 DATA:TCGA.B0.4848 DATA:TCGA.B0.4849 DATA:TCGA.B0.4852 DATA:TCGA.B0.4945 DATA:TCGA.B0.5075 DATA:TCGA.B0.5077 DATA:TCGA.B0.5080 DATA:TCGA.B0.5081 DATA:TCGA.B0.5085 DATA:TCGA.B0.5088 DATA:TCGA.B0.5092 DATA:TCGA.B0.5094 DATA:TCGA.B0.5095 DATA:TCGA.B0.5096 DATA:TCGA.B0.5097 DATA:TCGA.B0.5099 DATA:TCGA.B0.5100 DATA:TCGA.B0.5102 DATA:TCGA.B0.5104 DATA:TCGA.B0.5106 DATA:TCGA.B0.5107 DATA:TCGA.B0.5108 DATA:TCGA.B0.5109 DATA:TCGA.B0.5110 DATA:TCGA.B0.5113 DATA:TCGA.B0.5115 DATA:TCGA.B0.5116 DATA:TCGA.B0.5119 DATA:TCGA.B0.5120 DATA:TCGA.B0.5121 DATA:TCGA.B0.5399 DATA:TCGA.B0.5400 DATA:TCGA.B0.5402 DATA:TCGA.B0.5691 DATA:TCGA.B0.5692 DATA:TCGA.B0.5693 DATA:TCGA.B0.5694 DATA:TCGA.B0.5695 DATA:TCGA.B0.5696 DATA:TCGA.B0.5697 DATA:TCGA.B0.5698 DATA:TCGA.B0.5699 DATA:TCGA.B0.5701 DATA:TCGA.B0.5702 DATA:TCGA.B0.5703 DATA:TCGA.B0.5705 DATA:TCGA.B0.5706 DATA:TCGA.B0.5707 DATA:TCGA.B0.5709 DATA:TCGA.B0.5710 DATA:TCGA.B0.5711 DATA:TCGA.B0.5713 DATA:TCGA.B0.5812 DATA:TCGA.B2.3924 DATA:TCGA.B2.4098 DATA:TCGA.B2.4099 DATA:TCGA.B2.4101 DATA:TCGA.B2.5633 DATA:TCGA.B2.5635 DATA:TCGA.B2.5641 DATA:TCGA.B4.5377 DATA:TCGA.B8.4143 DATA:TCGA.B8.4146 DATA:TCGA.B8.4148 DATA:TCGA.B8.4151 DATA:TCGA.B8.4153 DATA:TCGA.B8.4154 DATA:TCGA.B8.4620 DATA:TCGA.B8.4621 DATA:TCGA.B8.4622 DATA:TCGA.B8.5158 DATA:TCGA.B8.5159 DATA:TCGA.B8.5163 DATA:TCGA.B8.5164 DATA:TCGA.B8.5165 DATA:TCGA.B8.5545 DATA:TCGA.B8.5546 DATA:TCGA.B8.5549 DATA:TCGA.B8.5550 DATA:TCGA.B8.5551 DATA:TCGA.B8.5552 DATA:TCGA.B8.5553 DATA:TCGA.BP.4158 DATA:TCGA.BP.4159 DATA:TCGA.BP.4160 DATA:TCGA.BP.4161 DATA:TCGA.BP.4162 DATA:TCGA.BP.4163 DATA:TCGA.BP.4164 DATA:TCGA.BP.4165 DATA:TCGA.BP.4166 DATA:TCGA.BP.4167 DATA:TCGA.BP.4169 DATA:TCGA.BP.4170 DATA:TCGA.BP.4173 DATA:TCGA.BP.4174 DATA:TCGA.BP.4176 DATA:TCGA.BP.4326 DATA:TCGA.BP.4329 DATA:TCGA.BP.4330 DATA:TCGA.BP.4331 DATA:TCGA.BP.4337 DATA:TCGA.BP.4338 DATA:TCGA.BP.4340 DATA:TCGA.BP.4341 DATA:TCGA.BP.4342 DATA:TCGA.BP.4343 DATA:TCGA.BP.4345 DATA:TCGA.BP.4346 DATA:TCGA.BP.4347 DATA:TCGA.BP.4349 DATA:TCGA.BP.4351 DATA:TCGA.BP.4352 DATA:TCGA.BP.4354 DATA:TCGA.BP.4355 DATA:TCGA.BP.4756 DATA:TCGA.BP.4758 DATA:TCGA.BP.4759 DATA:TCGA.BP.4761 DATA:TCGA.BP.4762 DATA:TCGA.BP.4763 DATA:TCGA.BP.4765 DATA:TCGA.BP.4766 DATA:TCGA.BP.4768 DATA:TCGA.BP.4770 DATA:TCGA.BP.4771 DATA:TCGA.BP.4774 DATA:TCGA.BP.4775 DATA:TCGA.BP.4777 DATA:TCGA.BP.4781 DATA:TCGA.BP.4782 DATA:TCGA.BP.4787 DATA:TCGA.BP.4789 DATA:TCGA.BP.4790 DATA:TCGA.BP.4797 DATA:TCGA.BP.4798 DATA:TCGA.BP.4799 DATA:TCGA.BP.4801 DATA:TCGA.BP.4803 DATA:TCGA.BP.4804 DATA:TCGA.BP.4807 DATA:TCGA.BP.4960 DATA:TCGA.BP.4961 DATA:TCGA.BP.4962 DATA:TCGA.BP.4963 DATA:TCGA.BP.4964 DATA:TCGA.BP.4967 DATA:TCGA.BP.4968 DATA:TCGA.BP.4970 DATA:TCGA.BP.4971 DATA:TCGA.BP.4972 DATA:TCGA.BP.4973 DATA:TCGA.BP.4974 DATA:TCGA.BP.4975 DATA:TCGA.BP.4976 DATA:TCGA.BP.4977 DATA:TCGA.BP.4981 DATA:TCGA.BP.4982 DATA:TCGA.BP.4983 DATA:TCGA.BP.4985 DATA:TCGA.BP.4986 DATA:TCGA.BP.4987 DATA:TCGA.BP.4988 DATA:TCGA.BP.4989 DATA:TCGA.BP.4991 DATA:TCGA.BP.4992 DATA:TCGA.BP.4993 DATA:TCGA.BP.4995 DATA:TCGA.BP.4998 DATA:TCGA.BP.4999 DATA:TCGA.BP.5000 DATA:TCGA.BP.5001 DATA:TCGA.BP.5004 DATA:TCGA.BP.5006 DATA:TCGA.BP.5007 DATA:TCGA.BP.5008 DATA:TCGA.BP.5009 DATA:TCGA.BP.5010 DATA:TCGA.BP.5168 DATA:TCGA.BP.5169 DATA:TCGA.BP.5170 DATA:TCGA.BP.5173 DATA:TCGA.BP.5174 DATA:TCGA.BP.5175 DATA:TCGA.BP.5176 DATA:TCGA.BP.5177 DATA:TCGA.BP.5178 DATA:TCGA.BP.5180 DATA:TCGA.BP.5182 DATA:TCGA.BP.5183 DATA:TCGA.BP.5184 DATA:TCGA.BP.5185 DATA:TCGA.BP.5186 DATA:TCGA.BP.5187 DATA:TCGA.BP.5189 DATA:TCGA.BP.5190 DATA:TCGA.BP.5191 DATA:TCGA.BP.5192 DATA:TCGA.BP.5194 DATA:TCGA.BP.5195 DATA:TCGA.BP.5196 DATA:TCGA.BP.5198 DATA:TCGA.BP.5199 DATA:TCGA.BP.5200 DATA:TCGA.BP.5201 DATA:TCGA.BP.5202 DATA:TCGA.CJ.4634 DATA:TCGA.CJ.4635 DATA:TCGA.CJ.4636

DATA:TCGA.CJ.4637 DATA:TCGA.CJ.4638 DATA:TCGA.CJ.4639

DATA:TCGA.CJ.4640 DATA:TCGA.CJ.4641 DATA:TCGA.CJ.4643

DATA:TCGA.CJ.4644 DATA:TCGA.CJ.4868 DATA:TCGA.CJ.4870

DATA:TCGA.CJ.4871 DATA:TCGA.CJ.4872 DATA:TCGA.CJ.4873

DATA:TCGA.CJ.4874 DATA:TCGA.CJ.4875 DATA:TCGA.CJ.4876

DATA:TCGA.CJ.4878 DATA:TCGA.CJ.4881 DATA:TCGA.CJ.4882

DATA:TCGA.CJ.4884 DATA:TCGA.CJ.4885 DATA:TCGA.CJ.4886

DATA:TCGA.CJ.4887 DATA:TCGA.CJ.4888 DATA:TCGA.CJ.4889

DATA:TCGA.CJ.4890 DATA:TCGA.CJ.4891 DATA:TCGA.CJ.4892

DATA:TCGA.CJ.4893 DATA:TCGA.CJ.4894 DATA:TCGA.CJ.4895

DATA:TCGA.CJ.4897 DATA:TCGA.CJ.4899 DATA:TCGA.CJ.4901

DATA:TCGA.CJ.4902 DATA:TCGA.CJ.4903 DATA:TCGA.CJ.4904

DATA:TCGA.CJ.4905 DATA:TCGA.CJ.4907 DATA:TCGA.CJ.4908

DATA:TCGA.CJ.4912 DATA:TCGA.CJ.4916 DATA:TCGA.CJ.4918

DATA:TCGA.CJ.4920 DATA:TCGA.CJ.4923 DATA:TCGA.CJ.5671

DATA:TCGA.CJ.5672 DATA:TCGA.CJ.5675 DATA:TCGA.CJ.5676

DATA:TCGA.CJ.5677 DATA:TCGA.CJ.5678 DATA:TCGA.CJ.5679

DATA:TCGA.CJ.5680 DATA:TCGA.CJ.5681 DATA:TCGA.CJ.5682

DATA:TCGA.CJ.5683 DATA:TCGA.CJ.5684 DATA:TCGA.CJ.5686

DATA:TCGA.CJ.6027 DATA:TCGA.CJ.6028 DATA:TCGA.CJ.6030

DATA:TCGA.CJ.6031 DATA:TCGA.CJ.6032 DATA:TCGA.CJ.6033

DATA:TCGA.CW.5580 DATA:TCGA.CW.5581 DATA:TCGA.CW.5583 DATA:TCGA.CW.5585 DATA:TCGA.CW.5588 DATA:TCGA.CW.5589 DATA:TCGA.CW.5591 DATA:TCGA.CW.6087 DATA:TCGA.CW.6090 DATA:TCGA.CW.6093 DATA:TCGA.CZ.4853 DATA:TCGA.CZ.4854 DATA:TCGA.CZ.4856 DATA:TCGA.CZ.4857 DATA:TCGA.CZ.4858 DATA:TCGA.CZ.4859 DATA:TCGA.CZ.4861 DATA:TCGA.CZ.4862 DATA:TCGA.CZ.4863 DATA:TCGA.CZ.4865 DATA:TCGA.CZ.4866 DATA:TCGA.CZ.5451 DATA:TCGA.CZ.5452 DATA:TCGA.CZ.5453 DATA:TCGA.CZ.5454 DATA:TCGA.CZ.5455 DATA:TCGA.CZ.5456 DATA:TCGA.CZ.5457 DATA:TCGA.CZ.5458 DATA:TCGA.CZ.5459 DATA:TCGA.CZ.5460 DATA:TCGA.CZ.5461 DATA:TCGA.CZ.5462 DATA:TCGA.CZ.5463 DATA:TCGA.CZ.5465 DATA:TCGA.CZ.5466 DATA:TCGA.CZ.5467 DATA:TCGA.CZ.5468 DATA:TCGA.CZ.5469 DATA:TCGA.CZ.5470 DATA:TCGA.CZ.5982 DATA:TCGA.CZ.5984 DATA:TCGA.CZ.5985 DATA:TCGA.CZ.5986 DATA:TCGA.CZ.5987 DATA:TCGA.CZ.5988 DATA:TCGA.CZ.5989 DATA:TCGA.DV.5565 DATA:TCGA.DV.5566 DATA:TCGA.DV.5568 DATA:TCGA.DV.5569 DATA:TCGA.DV.5574 DATA:TCGA.DV.5575 DATA:TCGA.DV.5576 DATA:TCGA.EU.5904 DATA:TCGA.EU.5905 DATA:TCGA.EU.5906 DATA:TCGA.EU.5907
