# Supplementary material for: NOD2 maybe a biomarker for the survival of kidney cancer patients
Source: Oncotarget. 2017 Oct 6;8(60):101489–99. doi: 10.18632/oncotarget.21547 (PMC5731890; doi:10.18632/oncotarget.21547)
Supplement: Supplementary file 4 [file oncotarget-08-101489-s004.doc]

#CENSORED:SURVIVAL_DAYS

214+ 2298+ 1795+ 1771 3050+ 1731+ 139 2790+ 1060+ 1728+ 1662+ 1567+ 1213+ 1186+ 1006+ 934+ 740+ 949+ 893+ 778+ 161+ 492 684+ 4+ 390+ 352+ 373+ 2043+ 3241+ 1508 2744+ 677+ 2392+ 471 1914+ 1309+ 395+ 377+ 824+ 2051+ 554+ 597 1857+ 3+ 650+ 1070 582+ 27+ 238+ 7+ 506+ 927+ 896+ 780+ 729+ 708+ 709+ 597+ 600+ 436+ 379+ 516+ 258 496+ 479+ 252+ 395 379+ 293 2135+ 2331 2029+ 1906+ 2060+ 1498 67+ 1251+ 1141+ 659+ 1110+ 842+ 616+ 510+ 618+ 641+ 1029+ 883+ 930+ 1049+ 379+ 309+ 1536 857+ 803+ 627+ 14+ 35+ 665+ 1338+ 671+ 361+ 585+ 524+ 5+ 452+ 532+ 374+ 2639+ 586+ 270 1938+ 697 1314 1454+ 658 1242+ 792+ 921+ 792+ 771+ 403+ 329 233+ 29+ 364+ 241 84+ 3760+ 1468 2948+ 2839+ 1364 1864+ 1533+ 1516+ 799+ 612+ 343 232+ 214+ 218+ 116+ 1486+ 414+ 3950+ 1093+ 1142+ 744+ 419+ 114+ 445+ 92+ 1240+ 2429+ 2460+ 1557 1856+ 2269+ 1040+ 578+ 757+ 325 398+ 73+ 36+ 13+ 122 1575+ 2765+ 336 1194+ 2173+ 765+ 624 786+ 768+ 547+ 702+ 514 2453+ 5925+ 393+ 112+ 841+ 32+ 378+ 546+ 392+ 341+ 496+ 2492+ 1770+ 2582+ 791+ 360+ 1726+ 2228+ 767+ 511+ 608+ 62+ 2609 2684 2941 2043 677+ 649+ 497+ 376+ 388+ 321 307+ 560+ 885+ 347+ 1043+ 845+ 924+ 841+ 616+ 536+ 369+ 947+ 890+ 471+ 3035+ 2649+ 1306 201 3015+ 2816+ 487 1539+ 600+ 540+ 428+ 1457+ 1504+ 1357+ 876+ 1076+ 823+ 363 578+ 1257+ 496 1266+ 805+ 1615+ 1331+ 548+ 797 3571+ 3152+ 3811+ 1136 1911+ 3010+ 2627+ 2167+ 933 1444+ 1981+ 657+ 3368+ 1562+ 965+ 791+ 1188+ 567+ 484+ 567+ 549+ 475+ 769+ 205+ 183+ 404+

SYMBOL:Gene

DATA:TCGA.2K.A9WE DATA:TCGA.2Z.A9J1 DATA:TCGA.2Z.A9J2

DATA:TCGA.2Z.A9J3 DATA:TCGA.2Z.A9J5 DATA:TCGA.2Z.A9J6

DATA:TCGA.2Z.A9J7 DATA:TCGA.2Z.A9J8 DATA:TCGA.2Z.A9JD DATA:TCGA.2Z.A9JE DATA:TCGA.2Z.A9JG DATA:TCGA.2Z.A9JI

DATA:TCGA.2Z.A9JJ DATA:TCGA.2Z.A9JK DATA:TCGA.2Z.A9JL

DATA:TCGA.2Z.A9JM DATA:TCGA.2Z.A9JN DATA:TCGA.2Z.A9JO DATA:TCGA.2Z.A9JP DATA:TCGA.2Z.A9JQ DATA:TCGA.2Z.A9JR

DATA:TCGA.2Z.A9JS DATA:TCGA.2Z.A9JT DATA:TCGA.4A.A93W

DATA:TCGA.4A.A93X DATA:TCGA.4A.A93Y DATA:TCGA.5P.A9JU

DATA:TCGA.5P.A9JV DATA:TCGA.5P.A9JW DATA:TCGA.5P.A9JY DATA:TCGA.5P.A9JZ DATA:TCGA.5P.A9K0 DATA:TCGA.5P.A9K2 DATA:TCGA.5P.A9K3 DATA:TCGA.5P.A9K6 DATA:TCGA.5P.A9K8 DATA:TCGA.5P.A9KA DATA:TCGA.5P.A9KC DATA:TCGA.5P.A9KE DATA:TCGA.5P.A9KH DATA:TCGA.A4.7286 DATA:TCGA.A4.7287 DATA:TCGA.A4.7288 DATA:TCGA.A4.7583 DATA:TCGA.A4.7584 DATA:TCGA.A4.7585 DATA:TCGA.A4.7732 DATA:TCGA.A4.7734 DATA:TCGA.A4.7828 DATA:TCGA.A4.7915 DATA:TCGA.A4.7996 DATA:TCGA.A4.7997 DATA:TCGA.A4.8098 DATA:TCGA.A4.8310 DATA:TCGA.A4.8311 DATA:TCGA.A4.8312 DATA:TCGA.A4.8515 DATA:TCGA.A4.8517 DATA:TCGA.A4.8518 DATA:TCGA.A4.8630 DATA:TCGA.A4.A48D DATA:TCGA.A4.A4ZT DATA:TCGA.A4.A57E DATA:TCGA.A4.A5DU DATA:TCGA.A4.A5XZ DATA:TCGA.A4.A5Y0 DATA:TCGA.A4.A5Y1 DATA:TCGA.A4.A6HP DATA:TCGA.AL.3466 DATA:TCGA.AL.3467 DATA:TCGA.AL.3468 DATA:TCGA.AL.3472 DATA:TCGA.AL.3473 DATA:TCGA.AL.7173 DATA:TCGA.AL.A5DJ DATA:TCGA.AT.A5NU DATA:TCGA.B1.5398 DATA:TCGA.B1.7332 DATA:TCGA.B1.A47M DATA:TCGA.B1.A47N DATA:TCGA.B1.A47O DATA:TCGA.B1.A654 DATA:TCGA.B1.A655 DATA:TCGA.B1.A656 DATA:TCGA.B1.A657 DATA:TCGA.B3.3925 DATA:TCGA.B3.3926 DATA:TCGA.B3.4103 DATA:TCGA.B3.4104 DATA:TCGA.B3.8121 DATA:TCGA.B3.A6W5 DATA:TCGA.B9.4113 DATA:TCGA.B9.4114 DATA:TCGA.B9.4115 DATA:TCGA.B9.4116 DATA:TCGA.B9.4117 DATA:TCGA.B9.4617 DATA:TCGA.B9.5155 DATA:TCGA.B9.5156 DATA:TCGA.B9.7268 DATA:TCGA.B9.A44B DATA:TCGA.B9.A5W7 DATA:TCGA.B9.A5W8 DATA:TCGA.B9.A5W9 DATA:TCGA.B9.A69E DATA:TCGA.B9.A8YH DATA:TCGA.B9.A8YI DATA:TCGA.BQ.5875

DATA:TCGA.BQ.5876 DATA:TCGA.BQ.5877 DATA:TCGA.BQ.5878 DATA:TCGA.BQ.5879 DATA:TCGA.BQ.5880 DATA:TCGA.BQ.5881

DATA:TCGA.BQ.5882 DATA:TCGA.BQ.5883 DATA:TCGA.BQ.5884

DATA:TCGA.BQ.5885 DATA:TCGA.BQ.5886 DATA:TCGA.BQ.5887 DATA:TCGA.BQ.5888 DATA:TCGA.BQ.5889 DATA:TCGA.BQ.5890 DATA:TCGA.BQ.5891 DATA:TCGA.BQ.5892 DATA:TCGA.BQ.5893 DATA:TCGA.BQ.5894 DATA:TCGA.BQ.7044 DATA:TCGA.BQ.7045 DATA:TCGA.BQ.7046 DATA:TCGA.BQ.7048 DATA:TCGA.BQ.7049

DATA:TCGA.BQ.7050 DATA:TCGA.BQ.7051 DATA:TCGA.BQ.7053 DATA:TCGA.BQ.7055 DATA:TCGA.BQ.7056 DATA:TCGA.BQ.7058 DATA:TCGA.BQ.7059 DATA:TCGA.BQ.7060 DATA:TCGA.BQ.7061 DATA:TCGA.BQ.7062 DATA:TCGA.DW.5560 DATA:TCGA.DW.5561 DATA:TCGA.DW.7834 DATA:TCGA.DW.7836 DATA:TCGA.DW.7837 DATA:TCGA.DW.7838 DATA:TCGA.DW.7839 DATA:TCGA.DW.7840 DATA:TCGA.DW.7841 DATA:TCGA.DW.7842 DATA:TCGA.DW.7963 DATA:TCGA.DZ.6131 DATA:TCGA.DZ.6132 DATA:TCGA.DZ.6133 DATA:TCGA.DZ.6134 DATA:TCGA.DZ.6135 DATA:TCGA.EV.5901 DATA:TCGA.EV.5902 DATA:TCGA.EV.5903 DATA:TCGA.F9.A4JJ

DATA:TCGA.F9.A7Q0 DATA:TCGA.F9.A7VF DATA:TCGA.F9.A8NY

DATA:TCGA.F9.A97G DATA:TCGA.G7.6789 DATA:TCGA.G7.6790 DATA:TCGA.G7.6792 DATA:TCGA.G7.6793 DATA:TCGA.G7.6795 DATA:TCGA.G7.6796 DATA:TCGA.G7.6797 DATA:TCGA.G7.7501 DATA:TCGA.G7.7502 DATA:TCGA.G7.A4TM DATA:TCGA.G7.A8LB DATA:TCGA.G7.A8LC DATA:TCGA.G7.A8LD DATA:TCGA.G7.A8LE DATA:TCGA.GL.6846 DATA:TCGA.GL.7773 DATA:TCGA.GL.7966 DATA:TCGA.GL.8500 DATA:TCGA.GL.A4EM DATA:TCGA.GL.A59R DATA:TCGA.GL.A59T DATA:TCGA.GL.A9DC DATA:TCGA.GL.A9DD DATA:TCGA.GL.A9DE DATA:TCGA.HE.7129 DATA:TCGA.HE.7130 DATA:TCGA.HE.A5NF DATA:TCGA.HE.A5NH DATA:TCGA.HE.A5NI DATA:TCGA.HE.A5NJ DATA:TCGA.HE.A5NK DATA:TCGA.HE.A5NL DATA:TCGA.IA.A40U DATA:TCGA.IA.A40X DATA:TCGA.IA.A40Y

DATA:TCGA.IA.A83S DATA:TCGA.IA.A83T DATA:TCGA.IA.A83V

DATA:TCGA.IA.A83W DATA:TCGA.IZ.8195 DATA:TCGA.IZ.8196

DATA:TCGA.IZ.A6M8 DATA:TCGA.IZ.A6M9 DATA:TCGA.J7.6720

DATA:TCGA.J7.8537 DATA:TCGA.J7.A8I2 DATA:TCGA.KV.A6GD

DATA:TCGA.KV.A6GE DATA:TCGA.KV.A74V DATA:TCGA.MH.A55W DATA:TCGA.MH.A55Z DATA:TCGA.MH.A560 DATA:TCGA.MH.A561 DATA:TCGA.MH.A562 DATA:TCGA.MH.A854 DATA:TCGA.MH.A855 DATA:TCGA.MH.A856 DATA:TCGA.MH.A857 DATA:TCGA.O9.A75Z DATA:TCGA.P4.A5E6 DATA:TCGA.P4.A5E7 DATA:TCGA.P4.A5E8 DATA:TCGA.P4.A5EA DATA:TCGA.P4.A5EB DATA:TCGA.P4.A5ED DATA:TCGA.P4.AAVL DATA:TCGA.P4.AAVM DATA:TCGA.PJ.A5Z8

DATA:TCGA.PJ.A5Z9 DATA:TCGA.Q2.A5QZ DATA:TCGA.SX.A71R DATA:TCGA.SX.A71S DATA:TCGA.SX.A71U DATA:TCGA.SX.A71V DATA:TCGA.SX.A71W DATA:TCGA.SX.A7SL DATA:TCGA.SX.A7SM DATA:TCGA.SX.A7SN DATA:TCGA.SX.A7SO DATA:TCGA.SX.A7SP DATA:TCGA.SX.A7SQ DATA:TCGA.SX.A7SR DATA:TCGA.SX.A7SS DATA:TCGA.SX.A7SU DATA:TCGA.UN.AAZ9 DATA:TCGA.UZ.A9PJ DATA:TCGA.UZ.A9PK DATA:TCGA.UZ.A9PL DATA:TCGA.UZ.A9PM DATA:TCGA.UZ.A9PN DATA:TCGA.UZ.A9PO DATA:TCGA.UZ.A9PP DATA:TCGA.UZ.A9PR DATA:TCGA.UZ.A9PS DATA:TCGA.UZ.A9PU DATA:TCGA.UZ.A9PV DATA:TCGA.UZ.A9PX DATA:TCGA.UZ.A9PZ

DATA:TCGA.UZ.A9Q0 DATA:TCGA.UZ.A9Q1 DATA:TCGA.V9.A7HT DATA:TCGA.WN.A9G9 DATA:TCGA.WN.AB4C DATA:TCGA.Y8.A894 DATA:TCGA.Y8.A895 DATA:TCGA.Y8.A896 DATA:TCGA.Y8.A897 DATA:TCGA.Y8.A898 DATA:TCGA.Y8.A8RY DATA:TCGA.Y8.A8RZ DATA:TCGA.Y8.A8S0 DATA:TCGA.Y8.A8S1
