# Supplementary material for: NOD2 maybe a biomarker for the survival of kidney cancer patients
Source: Oncotarget. 2017 Oct 6;8(60):101489–99. doi: 10.18632/oncotarget.21547 (PMC5731890; doi:10.18632/oncotarget.21547)
Supplement: Supplementary file 5 [file oncotarget-08-101489-s005.doc]

**#ORDINAL:AGE**

67 66 77 58 59 57 67 70 52 51 53 51 52 79 75 86 41 79 76 34 42 74 62 57 82 60 50 46 72 48 64 54 51 60 54 69 46 49 68 37 65 62 54 62 48 72 40 58 45 69 85 48 58 84 71 48 48 58 72 71 63 46 65 55 72 72 58 46 75 74 60 66 51 61 63 76 76 81 57 47 48 68 58 65 49 81 68 60 68 88 49 77 79 82 49 61 63 69 80 63 73 57 60 70 52 60 54 51 78 75 77 77 63 79 63 33 76 53 53 62 81 72 59 53 88 72 74 90 64 65 54 69 71 69 43 52 40 61 72 56 46 59 64 53 66 66 47 71 61 69 50 77 53 65 73 65 45 62 57 50 68 75 53 59 73 72 83 52 61 56 74 79 46 79 68 62 65 59 64 61 52 45 61 66 41 63 51 74 73 58 70 63 57 56 61 62 63 65 43 42 38 53 71 65 41 67 69 70 67 74 65 60 51 64 69 59 76 72 47 49 64 65 64 53 75 75 60 36 56 65 76 43 70 67 79 64 75 62 57 74 68 51 74 61 40 59 62 40 50 69 57 42 79 43 43 72 63 73 62 57 55 46 78 55 67 59 48 76 74 34 74 70 57 79 59 42 49 46 47 58 63 54 46 76 40 63 44 40 43 47 58 40 77 57 75 42 67 72 75 41 72 58 54 66 58 54 68 49 56 40 43 53 61 45 46 52 63 75 70 55 75 45 60 78 46 71 53 58 56 57 54 60 61 79 59 39 75 53 72 58 44 63 75 60 48 51 52 46 49 49 55 67 48 42 49 63 51 85 73 67 57 71 41 57 72 64 42 48 59 63 72 57 65 76 58 62 79 42 69 47 61 50 60 62 58 38 61 69 64 64 63 51 84 70 47 54 62 73 65 44 60 78 61 59 90 77 58 65 54 63 54 73 44 51 74 51 62 52 51 56 61 60 68 73 32 82 68 62 56 39 59 60 63 46 51 86 70 79 69 67 63 63 62 43 55 52 83 76 69 76 86 84 41 72 59 51 58 61 60 38 60 59 67 40 26 29 41 37 52 55

**#CENSORED:SURVIVAL_MONTHS**

37.33+ 47.87+ 0.53+ 39.67 24.5 49.77+ 49.7+ 37.67+ 50.27+ 49.23+ 36.83+ 39.53+ 25.03+ 26.17+ 23.53+ 49.5+ 22.13+ 31.47+ 36.57+ 46.17+ 30.33+ 18.7 47.5+ 43.57+ 42.87+ 51.97+ 10.63+ 29.07+ 35.13+ 25.87+ 24.5+ 11.13+ 43.77+ 21+ 18.9+ 19.13+ 24.2+ 20.53+ 111.43+ 29.47 85.53+ 74.1+ 67.23+ 61.77+ 73.07+ 69.53+ 68.1+ 58.13+ 47.43+ 42.63+ 49.97+ 49.37+ 39.67+ 29.1+ 22.77 1.53+ 38.93+ 31.7+ 28.43+ 12.3+ 1.33+ 3.37 1.43 4.63 2.57 3.53 28.87 19.23 1.4 3.63 65.97 7.9 6.07 2.17 19.97 44.57 6.7 3.27 7.13+ 15.9 47.23 0.6 5.57 52.9 45.67 33.97 17 6.1 40.97 15.13 55.23 29.47 10.23 79.5 69.67 41.27 45.93 27.8 54.6 6.77 57.43 10.67 10.43 66.17 39.97 26.4 29.4 2.3 37.37 71.5 21.23 43.87 11.4 12.07 34.83 7.37 25.63 18.77 15.3 11.1 8.17 2.23 5.03+ 52.8 16.17 63.77 92.1 91.7 53.23 30.9 5.4+ 19.53 18.67+ 0.73+ 3+ 3.43+ 1.6+ 1.83+ 6.2+ 6.33+ 22.57+ 0.33+ 72.67+ 92.7+ 49.57+ 61.73+ 15.97 34.73+ 31.47+ 37.83+ 72.7+ 80.83+ 42.53+ 8.73+ 111.17+ 80.47+ 78.57+ 31.9+ 85.1+ 81.27+ 62.13+ 77.47+ 5.93+ 6.47+ 1.67 3.67+ 3+ 0.5+ 7.13+ 10.5+ 8.6+ 6.37+ 5.6+ 9.7+ 5.83+ 0.23+ 1.27+ 0.53+ 4.7+ 5.53+ 0.37+ 0.23+ 23.63 17.03+ 12.63+ 9.3+ 13.47+ 8.5+ 0.63+ 7.53+ 7.17+ 6.03+ 3+ 4.43+ 0.73+ 0.13+ 0.77+ 0.27+ 8.27+ 0.47+ 6.47+ 6.57+ 0.5+ 7.3+ 0.47+ 112.57+ 86.67 96.03+ 91.53+ 102.47+ 94.63+ 33.07 98.2+ 0.43+ 90.6+ 23.37 78.1 63.1+ 62.63+ 65.17+ 55.67+ 98.8+ 54.13+ 3.6 28.17+ 62.93+ 37.77+ 21.5+ 15.33 0.07 91.53+ 18.73 52.97 75.2 63.73 55.53+ 50.53+ 49.77 45.57+ 12.4+ 32.3+ 11.47 12.5 34.47 31.77 12.47+ 73.6+ 77.47+ 74.97+ 6.07+ 44.77 42.33 72.8+ 48.7+ 13.33+ 62.53+ 10.97 5.37 62.8+ 61.4+ 57.7+ 69.33+ 11.8+ 61.8+ 15.97 49.63+ 37.03 20.67+ 36.9+ 11.13 37.77 37.47+ 6.77+ 48.63+ 7.03+ 88.67+ 72.4+ 64.5+ 59.5+ 61.13+ 62.07+ 62.37+ 6.8+ 58.17+ 59.8+ 14.4+ 49.57+ 50.03+ 46.13+ 7.33 47.77+ 54.4+ 15.13+ 36.57 33.77+ 47.07+ 31.7 26.17+ 37.47+ 27.57 3.9+ 34.8+ 16.67+ 5.87+ 43.57+ 45.7+ 31.03+ 42.2+ 18.77+ 39.23+ 37.53+ 28+ 38+ 35.7+ 36.4 29.23 48.77 6.4+ 80.37+ 1.8 75.23+ 31.07+ 53 9.77+ 63.73 63.07+ 49.83+ 38.83+ 36.73+ 13.53+ 27.4 33.67+ 32.2+ 23.8+ 13.6+ 24.93+ 33.93+ 20.1+ 45.13+ 29.37+ 31.7+ 0.97+ 60.67+ 47.2+ 64.13+ 74.23 14.37 76.93+ 66.6+ 55.37 59.73+ 11.2 21.5 85.13+ 80.73+ 47.83+ 75.27+ 76.1+ 78.43+ 65.17+ 72.87+ 67.1+ 62.77+ 33.1+ 70.83+ 65.03+ 31.03 52.2 64.83+ 69.5+ 27.27 50.67+ 24.97+ 28.03 40 60.27+ 50.9+ 57.1 48.33+ 50.63+ 51.97+ 59.73+ 49.83+ 49.93+ 51+ 55.2+ 45.73+ 3.07 4.63 19.07 64.73+ 73 80.97+ 85.83+ 26.07 19.1 22.6 25.6 18.37 62.77+ 62.97+ 74.33+ 67.93+ 54 61.83+ 54.17 76.6 63.5+ 84.93+ 7.47 65.47 93.3+ 82.97+ 5.47 86.97+ 74.2+ 79.23+ 35.83 75.67+ 1.37 77.03+ 76.93+ 81+ 19 25.8+ 46.8 0.6+ 47.73 64.77+ 59.57+ 6.83 14.83 61.43+ 56.33+ 94.33 5.5 58.93+ 51.87+ 0.83+ 24.07 18.7 51.57+ 51.93+ 47.67+ 11 10.37 22.07+ 49.73+ 45.9+ 2.43 1.97 31.5 68.07+ 49.7+ 54.3+ 12.43+ 14.5+ 23.1+ 53.3+ 44.3+ 46.6+ 30.3+ 12.33+ 11.83+ 37.67+ 24.1+ 33.5+ 24.23

**#CLASS:GRADE**

G3 G3 G2 G2 G3 G3 G2 G2 G1 G2 G1 G3 G2 G2 G2 G2 G4 G3 G2 G2 G2 G3 G3 G2 G2 G2 G2 G2 G3 G2 G2 G3 G2 G3 G2 G3 G2 G2 G2 G3 GX G2 G2 G3 GX G2 G2 G3 GX G3 G2 G3 G2 G3 G3 G3 G3 G2 G2 GX [Not Available] G4 G3 G3 G4 G4 G3 G4 G4 G4 G4 G3 G4 G4 G4 G3 G2 G3 G2 G3 G3 G3 G3 G4 G3 G3 G3 G4 G3 G2 G3 G4 G3 G2 G3 G3 G3 G3 G2 G3 G4 G3 G3 G2 G2 G3 G3 G3 G2 G2 G2 G3 G3 G2 G3 G3 G3 G3 G3 G2 G3 GX G2 G3 G3 G3 G3 G2 G2 G4 G2 G4 G2 G2 G3 G3 G2 G2 G2 G2 G2 G4 G4 G1 G3 G3 G2 G3 G2 G4 G2 G3 G2 G4 G3 G2 G2 G3 G2 G3 G3 G3 G3 G2 G2 G2 G3 G3 G2 G2 G2 G2 G3 G3 G3 G2 G2 G1 G2 G2 G2 G2 G1 G3 G2 G3 G2 G3 G2 G2 G2 G3 G3 G4 G3 G2 G3 G3 G2 G2 G2 G3 G3 G3 G2 G2 G2 G2 G2 G3 G2 G3 G2 G1 G3 G2 G2 G2 G3 G3 G2 G2 G2 G2 G2 G2 G2 G2 G3 G3 G4 G3 G2 G2 G3 G3 G2 G3 G3 G2 G2 G2 G4 G2 G4 G4 G2 G2 G2 G2 G4 G3 G2 G2 G3 G2 G2 G4 G4 G2 G2 G3 G3 G2 G2 G4 G2 G2 G2 G3 G4 G3 G2 G3 G2 G3 G3 G3 G2 G2 G3 G2 G2 G2 G3 G2 G3 G3 G3 G3 G4 G3 G3 G3 G3 G3 G4 G4 G3 G2 G2 G3 G2 G4 G3 G3 G3 G3 G2 G3 G2 G3 G2 G2 G2 G3 G4 G2 G4 G2 G2 G2 G3 G2 G3 G4 G2 G2 G3 G3 G2 G4 G3 G2 G2 G2 G2 G2 G3 G4 G4 G4 G2 G2 G3 G3 G4 G4 G2 G4 G4 G3 G3 G3 G2 G4 G4 G3 G3 G3 G3 G2 G3 G3 G3 G3 G3 G3 G4 G4 G4 G4 G2 G3 G3 G4 G3 G2 G4 G3 G3 G3 G3 G2 G3 G2 G3 G3 G4 G2 G4 G3 G3 G3 G3 G4 G3 G4 G4 G3 G4 G3 G2 G3 G4 G4 G4 G3 G3 G3 G4 G3 G3 G2 G3 G2 G2 G2 G3 G2 G4 G2 G3 G1 G4 G2 G2 G2 G3 G4 G2 G4 G2 G2 G3 G3 G2 G3 G2 G2 G2 G4 G4 G3 G2 G4 G3 G2 G2 G2 G4 G4 G2 G3 G2 G3 G2 G3 G2 G2 G2 G2 G2 G2 G2 G2 G2 G2 G2 G2

**#CLASS:PATHOLOGY**

T1b T3b T3b T1 T1b T2 T2 T1b T1b T1a T1b T1b T1a T1b T1b T1 T2a T2 T1b T1b T2a T3a T2 T1a T1a T1a T2 T1a T1b T1b T3 T1b T1b T1 T1 T1b T1a T1a T1 T3a T1a T3b T2 T2 T2 T1b T2 T1a T2 T3a T1a T2 T2 T1b T3b T2 T1b T1a T1a T1b T1a T4 T4 T2 T3a T3b T3a T3b T4 T4 T4 T3a T3a T3a T3a T3a T3b T3b T3a T3a T3a T3b T4 T3a T2 T3c T2 T3b T3b T1a T1a T3b T2 T1b T1a T3b T1b T1b T1b T2 T3a T3a T3a T3a T3a T3a T3b T3a T2 T1a T3a T1a T3a T3b T1a T3a T3a T1b T1a T3b T3a T3a T3b T1 T3b T3a T1 T1 T1a T2 T3a T3b T1a T3a T2 T3b T1b T1b T1a T1b T1b T3b T4 T1b T1a T3b T1b T3a T1b T3a T1a T1b T1 T3b T1b T1 T2 T3a T1b T3b T2 T3b T1b T2 T1b T1b T1a T2a T1b T1b T1a T1a T3 T1a T3 T1 T3b T1 T1 T1b T3 T1 T2 T3a T1b T1a T3a T3a T1a T1a T3a T1b T3a T3a T1a T2a T3a T3a T1a T1a T1b T1b T3a T1b T1b T1b T1b T1b T3a T1b T1b T3a T3a T1b T3a T3a T2 T1b T2 T2 T1b T1a T1b T1b T2 T3a T3a T3a T3a T3a T3b T1b T1b T3a T2 T3a T1a T3b T3b T3b T1a T3a T3b T1 T4 T3a T1b T1a T1a T1a T3a T1a T1a T1a T1a T1a T1a T4 T3a T1a T1a T1a T1a T1a T1a T3a T1a T1a T1a T3b T3b T3b T1a T3a T1b T1a T1b T2 T1a T2 T1b T1a T1a T3a T1b T1a T1a T3a T3a T3a T3a T1b T1a T1b T3a T1b T3a T3a T1a T1b T1a T3a T1a T1b T1a T1a T1b T1a T1a T1b T1b T1a T1a T2 T1a T1b T3a T1a T1b T1a T1a T1a T1a T1a T1a T3a T1a T1b T1a T3a T1a T1b T1a T3a T1a T1a T1a T1a T3b T2 T2 T3b T3a T1b T1b T3a T2b T3a T2 T3a T3a T2b T3a T3a T2 T3a T1b T3a T1b T3a T2b T3a T3a T3a T3a T3a T1a T3a T3a T1a T3a T3c T1b T1b T3a T3a T3a T1b T4 T3b T3a T1b T3a T1a T3b T1a T2 T3a T3a T1b T3a T1a T1a T2a T3b T3a T2b T3b T3a T3a T3a T1b T3a T1b T1b T1a T3a T1a T1b T2 T3a T3a T1b T1a T3b T3b T3b T1a T3a T3a T3a T1b T1b T1a T3a T1a T1b T1b T3a T2 T1 T4 T2 T1b T3b T2 T1a T1 T2 T2 T2 T3b T3a T3a T3b T1b T1b T2 T3b T3b T3a T3b T2 T2 T1a T1b T2 T1 T3b T1b T2 T1a T1a T1a T1a T1a T1a T1a T1a T1a

**#CLASS:STAGE**

Stage I Stage II Stage II Stage I Stage I Stage II Stage II Stage I Stage I Stage I Stage I Stage I Stage I Stage I Stage I Stage I Stage II Stage II Stage III Stage I Stage II Stage III Stage II Stage I Stage I Stage I Stage II Stage I Stage I Stage I Stage III Stage I Stage I Stage I Stage I Stage I Stage I Stage I Stage I Stage III Stage I Stage III Stage II Stage III Stage II Stage I Stage IV Stage I Stage II Stage I Stage I Stage II Stage II Stage I Stage III Stage II Stage I Stage I Stage I Stage I Stage I Stage IV Stage IV Stage IV Stage III Stage III Stage III Stage IV Stage IV Stage IV Stage IV Stage IV Stage IV Stage III Stage III Stage IV Stage III Stage IV Stage IV Stage III Stage III Stage III Stage IV Stage III Stage II Stage III Stage IV Stage IV Stage III Stage I Stage I Stage III Stage IV Stage I Stage I Stage IV Stage I Stage I Stage I Stage IV Stage III Stage III Stage IV Stage IV Stage IV Stage IV Stage III Stage III Stage II Stage I Stage III Stage I Stage IV Stage III Stage I Stage IV Stage III Stage I Stage IV Stage IV Stage III Stage III Stage III Stage I Stage III Stage III Stage I Stage I Stage I Stage IV Stage III Stage IV Stage I Stage III Stage III Stage III Stage I Stage I Stage I Stage I Stage I Stage III Stage IV Stage I Stage I Stage III Stage I Stage III Stage I Stage III Stage I Stage I Stage I Stage III Stage I Stage I Stage II Stage III Stage I Stage III Stage IV Stage III Stage I Stage II Stage I Stage I Stage I Stage II Stage I Stage I Stage I Stage I Stage III Stage I Stage IV Stage I Stage III Stage I Stage I Stage I Stage III Stage I Stage II Stage IV Stage I Stage I Stage III Stage III Stage I Stage I Stage III Stage I Stage IV Stage III Stage I Stage II Stage III Stage III Stage I Stage I Stage I Stage I Stage III Stage I Stage I Stage I Stage I Stage I Stage III Stage I Stage I Stage III Stage III Stage I Stage III Stage III Stage II Stage I Stage II Stage II Stage I Stage I Stage I Stage I Stage II Stage III Stage III Stage III Stage III Stage IV Stage III Stage I Stage I Stage III Stage II Stage III Stage I Stage III Stage III Stage III Stage I Stage III Stage IV Stage I Stage IV Stage III Stage I Stage I Stage I Stage I Stage III Stage I Stage I Stage I Stage I Stage I Stage I Stage IV Stage IV Stage I Stage I Stage I Stage I Stage I Stage I Stage IV Stage I Stage I Stage I Stage III Stage III Stage III Stage I Stage III Stage I Stage I Stage I Stage II Stage I Stage II Stage I Stage I Stage I Stage III Stage I Stage I Stage III Stage III Stage III Stage III Stage IV Stage I Stage I Stage I Stage III Stage I Stage III Stage III Stage I Stage I Stage I Stage III Stage I Stage I Stage I Stage I Stage I Stage I Stage I Stage I Stage I Stage I Stage I Stage II Stage I Stage I Stage III Stage I Stage I Stage I Stage I Stage I Stage I Stage I Stage I Stage IV Stage I Stage I Stage I Stage III Stage I Stage I Stage I Stage III Stage I Stage I Stage I Stage I Stage III Stage II Stage II Stage IV Stage III Stage I Stage I Stage III Stage IV Stage IV Stage II Stage III Stage IV Stage II Stage IV Stage IV Stage III Stage IV Stage I Stage III Stage I Stage IV Stage II Stage III Stage III Stage III Stage III Stage IV Stage I Stage IV Stage IV Stage I Stage IV Stage III Stage I Stage I Stage III Stage IV Stage III Stage I Stage IV Stage III Stage III Stage I Stage IV Stage I Stage III Stage I Stage II Stage III Stage IV Stage I Stage IV Stage I Stage I Stage II Stage III Stage IV Stage IV Stage III Stage IV Stage IV Stage IV Stage I Stage III Stage I Stage I Stage I Stage IV Stage I Stage I Stage II Stage IV Stage IV Stage I Stage I Stage III Stage IV Stage III Stage I Stage IV Stage IV Stage IV Stage I Stage I Stage I Stage III Stage I Stage I Stage I Stage IV Stage II Stage I Stage IV Stage II Stage I Stage III Stage II Stage I Stage I Stage II Stage II Stage IV Stage IV Stage III Stage III Stage IV Stage IV Stage IV Stage III Stage IV Stage III Stage III Stage IV Stage II Stage II Stage I Stage I Stage II Stage I Stage IV Stage I Stage II Stage I Stage I Stage I Stage I Stage I Stage I Stage I Stage I Stage I

#CLASS:DEATH

0 0 0 1 1 0 0 0 0 0 0 0 0 0 0 0 0 0 0 0 0 1 0 0 0 0 0 0 0 0 0 0 0 0 0 0 0 0 0 1 0 0 0 0 0 0 0 0 0 0 0 0 0 0 1 0 0 0 0 0 0 1 1 1 1 1 1 1 1 1 1 1 1 1 1 1 1 1 0 1 1 1 1 1 1 1 1 1 1 1 1 1 1 1 1 1 1 1 1 1 1 1 1 1 1 1 1 1 1 1 1 1 1 1 1 1 1 1 1 1 1 1 0 1 1 1 1 1 1 1 0 1 0 0 0 0 0 0 0 0 0 0 0 0 0 0 0 1 0 0 0 0 0 0 0 0 0 0 0 0 0 0 0 0 0 1 0 0 0 0 0 0 0 0 0 0 0 0 0 0 0 0 0 1 0 0 0 0 0 0 0 0 0 0 0 0 0 0 0 0 0 0 0 0 0 0 0 1 0 0 0 0 1 0 0 0 1 1 0 0 0 0 0 0 1 0 0 0 0 1 1 0 1 1 1 1 0 0 1 0 0 0 1 1 1 1 0 0 0 0 0 1 1 0 0 0 0 1 1 0 0 0 0 0 0 1 0 1 0 0 1 1 0 0 0 0 0 0 0 0 0 0 0 0 0 0 0 0 0 0 1 0 0 0 1 0 0 1 0 0 1 0 0 0 0 0 0 0 0 0 0 0 0 0 0 1 1 1 0 0 1 0 0 1 0 1 0 0 0 0 0 1 0 0 0 0 0 0 0 0 0 0 0 0 0 0 1 1 0 0 1 0 1 1 0 0 0 0 0 0 0 0 0 0 0 0 0 1 1 0 0 1 0 0 1 1 0 0 1 0 0 0 0 0 0 0 0 0 1 1 1 0 1 0 0 1 1 1 1 1 0 0 0 0 1 0 1 1 0 0 1 1 0 0 1 0 0 0 1 0 1 0 0 0 1 0 1 0 1 0 0 1 1 0 0 1 1 0 0 0 1 1 0 0 0 1 1 0 0 0 1 1 1 0 0 0 0 0 1 0 0 0 0 0 0 0 0 0 0 1

**ID:Entrez SYMBOL:Gene_Symbol**

Sample:TCGA.A3.3306.01A.01R.0864.07 Sample:TCGA.A3.3307.01A.01R.0864.07 Sample:TCGA.A3.3308.01A.02R.1325.07 Sample:TCGA.A3.3311.01A.02R.1325.07 Sample:TCGA.A3.3313.01A.02R.1325.07 Sample:TCGA.A3.3316.01A.01R.0864.07 Sample:TCGA.A3.3317.01A.02R.1325.07 Sample:TCGA.A3.3319.01A.02R.1325.07 Sample:TCGA.A3.3320.01A.02R.1325.07 Sample:TCGA.A3.3322.01A.02R.1325.07 Sample:TCGA.A3.3323.01A.02R.1325.07 Sample:TCGA.A3.3324.01A.02R.1325.07 Sample:TCGA.A3.3325.01A.01R.0864.07 Sample:TCGA.A3.3328.01A.01R.0864.07 Sample:TCGA.A3.3329.01A.01R.0864.07 Sample:TCGA.A3.3331.01A.02R.1325.07 Sample:TCGA.A3.3335.01A.01R.0864.07 Sample:TCGA.A3.3343.01A.01R.0864.07 Sample:TCGA.A3.3347.01A.02R.1325.07 Sample:TCGA.A3.3349.01A.01R.1188.07 Sample:TCGA.A3.3351.01A.02R.1325.07 Sample:TCGA.A3.3352.01A.01R.0864.07 Sample:TCGA.A3.3357.01A.02R.1420.07 Sample:TCGA.A3.3358.01A.01R.1541.07 Sample:TCGA.A3.3359.01A.01R.0864.07 Sample:TCGA.A3.3362.01A.02R.1325.07 Sample:TCGA.A3.3363.01A.01R.0864.07 Sample:TCGA.A3.3365.01A.01R.0864.07 Sample:TCGA.A3.3367.01A.02R.1420.07 Sample:TCGA.A3.3370.01A.02R.1420.07 Sample:TCGA.A3.3372.01A.02R.1325.07 Sample:TCGA.A3.3373.01A.02R.1420.07 Sample:TCGA.A3.3374.01A.02R.1325.07 Sample:TCGA.A3.3378.01A.02R.1325.07 Sample:TCGA.A3.3380.01A.01R.0864.07 Sample:TCGA.A3.3382.01A.02R.1325.07 Sample:TCGA.A3.3385.01A.02R.1420.07 Sample:TCGA.A3.3387.01A.01R.1541.07 Sample:TCGA.AK.3425.01A.02R.1277.07 Sample:TCGA.AK.3426.01A.02R.1325.07 Sample:TCGA.AK.3427.01A.01R.0864.07 Sample:TCGA.AK.3428.01A.02R.1277.07 Sample:TCGA.AK.3429.01A.02R.1325.07 Sample:TCGA.AK.3431.01A.02R.1277.07 Sample:TCGA.AK.3433.01A.02R.1277.07 Sample:TCGA.AK.3434.01A.02R.1277.07 Sample:TCGA.AK.3436.01A.02R.1325.07 Sample:TCGA.AK.3440.01A.02R.1277.07 Sample:TCGA.AK.3443.01A.02R.1325.07 Sample:TCGA.AK.3445.01A.02R.1277.07 Sample:TCGA.AK.3450.01A.02R.1277.07 Sample:TCGA.AK.3451.01A.02R.1188.07 Sample:TCGA.AK.3453.01A.02R.1277.07 Sample:TCGA.AK.3454.01A.02R.1277.07 Sample:TCGA.AK.3455.01A.01R.0864.07 Sample:TCGA.AK.3456.01A.02R.1325.07 Sample:TCGA.AK.3458.01A.01R.1503.07 Sample:TCGA.AK.3460.01A.02R.1277.07 Sample:TCGA.AK.3461.01A.02R.1277.07 Sample:TCGA.AK.3465.01A.02R.1325.07 Sample:TCGA.AS.3777.01A.01R.0864.07 Sample:TCGA.B0.4688.01A.01R.1277.07 Sample:TCGA.B0.4690.01A.01R.1277.07 Sample:TCGA.B0.4691.01A.01R.1277.07 Sample:TCGA.B0.4693.01A.01R.1277.07 Sample:TCGA.B0.4694.01A.01R.1277.07 Sample:TCGA.B0.4696.01A.01R.1277.07 Sample:TCGA.B0.4697.01A.01R.1277.07 Sample:TCGA.B0.4698.01A.01R.1503.07 Sample:TCGA.B0.4699.01A.01R.1277.07 Sample:TCGA.B0.4700.01A.02R.1541.07 Sample:TCGA.B0.4701.01A.01R.1277.07 Sample:TCGA.B0.4703.01A.01R.1277.07 Sample:TCGA.B0.4706.01A.01R.1503.07 Sample:TCGA.B0.4707.01A.01R.1277.07 Sample:TCGA.B0.4712.01A.01R.1503.07 Sample:TCGA.B0.4713.01A.01R.1277.07 Sample:TCGA.B0.4714.01A.01R.1277.07 Sample:TCGA.B0.4718.01A.01R.1277.07 Sample:TCGA.B0.4810.01A.01R.1503.07 Sample:TCGA.B0.4811.01A.01R.1503.07 Sample:TCGA.B0.4813.01A.01R.1277.07 Sample:TCGA.B0.4814.01A.01R.1277.07 Sample:TCGA.B0.4815.01A.01R.1503.07 Sample:TCGA.B0.4816.01A.01R.1503.07 Sample:TCGA.B0.4817.01A.01R.1277.07 Sample:TCGA.B0.4818.01A.01R.1503.07 Sample:TCGA.B0.4819.01A.01R.1277.07 Sample:TCGA.B0.4821.01A.01R.1503.07 Sample:TCGA.B0.4823.01A.02R.1420.07 Sample:TCGA.B0.4824.01A.01R.1277.07 Sample:TCGA.B0.4827.01A.02R.1420.07 Sample:TCGA.B0.4828.01A.01R.1277.07 Sample:TCGA.B0.4833.01A.01R.1305.07 Sample:TCGA.B0.4834.01A.01R.1305.07 Sample:TCGA.B0.4836.01A.01R.1305.07 Sample:TCGA.B0.4837.01A.01R.1305.07 Sample:TCGA.B0.4838.01A.01R.1305.07 Sample:TCGA.B0.4839.01A.01R.1305.07 Sample:TCGA.B0.4841.01A.01R.1277.07 Sample:TCGA.B0.4842.01A.02R.1420.07 Sample:TCGA.B0.4843.01A.01R.1277.07 Sample:TCGA.B0.4844.01A.01R.1277.07 Sample:TCGA.B0.4845.01A.01R.1277.07 Sample:TCGA.B0.4846.01A.01R.1277.07 Sample:TCGA.B0.4847.01A.01R.1277.07 Sample:TCGA.B0.4848.01A.01R.1277.07 Sample:TCGA.B0.4849.01A.01R.1277.07 Sample:TCGA.B0.4852.01A.01R.1503.07 Sample:TCGA.B0.4945.01A.01R.1420.07 Sample:TCGA.B0.5075.01A.01R.1334.07 Sample:TCGA.B0.5077.01A.01R.1334.07 Sample:TCGA.B0.5080.01A.01R.1503.07 Sample:TCGA.B0.5081.01A.01R.1334.07 Sample:TCGA.B0.5083.01A.02R.1420.07 Sample:TCGA.B0.5084.01A.01R.1334.07 Sample:TCGA.B0.5085.01A.01R.1334.07 Sample:TCGA.B0.5088.01A.01R.1334.07 Sample:TCGA.B0.5092.01A.01R.1420.07 Sample:TCGA.B0.5094.01A.01R.1420.07 Sample:TCGA.B0.5095.01A.01R.1420.07 Sample:TCGA.B0.5096.01A.01R.1420.07 Sample:TCGA.B0.5097.01A.01R.1420.07 Sample:TCGA.B0.5098.01A.01R.1420.07 Sample:TCGA.B0.5099.01A.01R.1420.07 Sample:TCGA.B0.5100.01A.01R.1420.07 Sample:TCGA.B0.5102.01A.01R.1420.07 Sample:TCGA.B0.5104.01A.01R.1420.07 Sample:TCGA.B0.5106.01A.01R.1420.07 Sample:TCGA.B0.5107.01A.01R.1420.07 Sample:TCGA.B0.5108.01A.01R.1420.07 Sample:TCGA.B0.5109.01A.02R.1420.07 Sample:TCGA.B0.5110.01A.01R.1420.07 Sample:TCGA.B0.5113.01A.01R.1420.07 Sample:TCGA.B0.5115.01A.01R.1420.07 Sample:TCGA.B0.5116.01A.02R.1420.07 Sample:TCGA.B0.5117.01A.01R.1420.07 Sample:TCGA.B0.5119.01A.02R.1420.07 Sample:TCGA.B0.5120.01A.01R.1420.07 Sample:TCGA.B0.5121.01A.02R.1420.07 Sample:TCGA.B0.5399.01A.01R.1503.07 Sample:TCGA.B0.5400.01A.01R.1503.07 Sample:TCGA.B0.5402.01A.01R.1503.07 Sample:TCGA.B0.5690.01A.11R.1541.07 Sample:TCGA.B0.5691.01A.11R.1541.07 Sample:TCGA.B0.5692.01A.11R.1541.07 Sample:TCGA.B0.5693.01A.11R.1541.07 Sample:TCGA.B0.5694.01A.11R.1541.07 Sample:TCGA.B0.5695.01A.11R.1541.07 Sample:TCGA.B0.5696.01A.11R.1541.07 Sample:TCGA.B0.5697.01A.11R.1541.07 Sample:TCGA.B0.5698.01A.11R.1672.07 Sample:TCGA.B0.5699.01A.11R.1541.07 Sample:TCGA.B0.5701.01A.11R.1541.07 Sample:TCGA.B0.5703.01A.11R.1541.07 Sample:TCGA.B0.5705.01A.11R.1541.07 Sample:TCGA.B0.5706.01A.11R.1541.07 Sample:TCGA.B0.5709.01A.11R.1541.07 Sample:TCGA.B0.5710.01A.11R.1672.07 Sample:TCGA.B0.5711.01A.11R.1672.07 Sample:TCGA.B0.5712.01A.11R.1672.07 Sample:TCGA.B0.5713.01A.11R.1672.07 Sample:TCGA.B0.5812.01A.11R.1672.07 Sample:TCGA.B2.3923.01A.02R.1325.07 Sample:TCGA.B2.3924.01A.02R.1325.07 Sample:TCGA.B2.4098.01A.02R.1325.07 Sample:TCGA.B2.4099.01A.02R.1188.07 Sample:TCGA.B2.4101.01A.02R.1277.07 Sample:TCGA.B2.4102.01A.02R.1325.07 Sample:TCGA.B2.5633.01A.01R.1541.07 Sample:TCGA.B2.5635.01A.01R.1541.07 Sample:TCGA.B2.5636.01A.02R.1541.07 Sample:TCGA.B2.5639.01A.01R.1541.07 Sample:TCGA.B2.5641.01A.01R.1541.07 Sample:TCGA.B4.5377.01A.01R.1503.07 Sample:TCGA.B4.5378.01A.01R.1503.07 Sample:TCGA.B4.5832.01A.11R.1672.07 Sample:TCGA.B4.5834.01A.11R.1672.07 Sample:TCGA.B4.5835.01A.11R.1672.07 Sample:TCGA.B4.5836.01A.11R.1672.07 Sample:TCGA.B4.5838.01A.11R.1672.07 Sample:TCGA.B4.5843.01A.11R.1672.07 Sample:TCGA.B4.5844.01A.11R.1672.07 Sample:TCGA.B8.4143.01A.01R.1188.07 Sample:TCGA.B8.4146.01B.11R.1672.07 Sample:TCGA.B8.4148.01A.02R.1325.07 Sample:TCGA.B8.4151.01A.01R.1188.07 Sample:TCGA.B8.4153.01B.11R.1672.07 Sample:TCGA.B8.4154.01A.01R.1188.07 Sample:TCGA.B8.4619.01A.02R.1325.07 Sample:TCGA.B8.4620.01A.02R.1325.07 Sample:TCGA.B8.4621.01A.01R.1503.07 Sample:TCGA.B8.4622.01A.02R.1277.07 Sample:TCGA.B8.5158.01A.01R.1420.07 Sample:TCGA.B8.5159.01A.01R.1420.07 Sample:TCGA.B8.5162.01A.01R.1420.07 Sample:TCGA.B8.5163.01A.01R.1420.07 Sample:TCGA.B8.5164.01A.01R.1420.07 Sample:TCGA.B8.5165.01A.01R.1420.07 Sample:TCGA.B8.5545.01A.01R.1672.07 Sample:TCGA.B8.5546.01A.01R.1541.07 Sample:TCGA.B8.5549.01A.01R.1541.07 Sample:TCGA.B8.5550.01A.01R.1541.07 Sample:TCGA.B8.5551.01A.01R.1541.07 Sample:TCGA.B8.5552.01B.11R.1672.07 Sample:TCGA.B8.5553.01A.01R.1541.07 Sample:TCGA.BP.4158.01A.02R.1289.07 Sample:TCGA.BP.4159.01A.02R.1289.07 Sample:TCGA.BP.4160.01A.02R.1289.07 Sample:TCGA.BP.4161.01A.02R.1325.07 Sample:TCGA.BP.4162.01A.02R.1325.07 Sample:TCGA.BP.4163.01A.02R.1325.07 Sample:TCGA.BP.4164.01A.02R.1325.07 Sample:TCGA.BP.4165.01A.02R.1289.07 Sample:TCGA.BP.4166.01A.02R.1289.07 Sample:TCGA.BP.4167.01A.02R.1325.07 Sample:TCGA.BP.4169.01A.02R.1289.07 Sample:TCGA.BP.4170.01A.02R.1289.07 Sample:TCGA.BP.4173.01A.02R.1289.07 Sample:TCGA.BP.4174.01A.02R.1289.07 Sample:TCGA.BP.4176.01A.02R.1289.07 Sample:TCGA.BP.4177.01A.02R.1420.07 Sample:TCGA.BP.4325.01A.02R.1289.07 Sample:TCGA.BP.4326.01A.01R.1289.07 Sample:TCGA.BP.4327.01A.01R.1289.07 Sample:TCGA.BP.4329.01A.02R.1289.07 Sample:TCGA.BP.4330.01A.01R.1289.07 Sample:TCGA.BP.4332.01A.01R.1289.07 Sample:TCGA.BP.4334.01A.01R.1289.07 Sample:TCGA.BP.4335.01A.01R.1289.07 Sample:TCGA.BP.4337.01A.01R.1289.07 Sample:TCGA.BP.4338.01A.01R.1289.07 Sample:TCGA.BP.4340.01A.01R.1289.07 Sample:TCGA.BP.4341.01A.01R.1289.07 Sample:TCGA.BP.4342.01A.01R.1289.07 Sample:TCGA.BP.4343.01A.02R.1289.07 Sample:TCGA.BP.4344.01A.01R.1289.07 Sample:TCGA.BP.4345.01A.01R.1289.07 Sample:TCGA.BP.4346.01A.01R.1289.07 Sample:TCGA.BP.4347.01A.01R.1289.07 Sample:TCGA.BP.4349.01A.01R.1289.07 Sample:TCGA.BP.4351.01A.01R.1289.07 Sample:TCGA.BP.4352.01A.01R.1289.07 Sample:TCGA.BP.4353.01A.02R.1289.07 Sample:TCGA.BP.4354.01A.02R.1289.07 Sample:TCGA.BP.4355.01A.01R.1289.07 Sample:TCGA.BP.4756.01A.01R.1289.07 Sample:TCGA.BP.4758.01A.01R.1289.07 Sample:TCGA.BP.4759.01A.01R.1289.07 Sample:TCGA.BP.4760.01A.02R.1420.07 Sample:TCGA.BP.4761.01A.01R.1289.07 Sample:TCGA.BP.4762.01A.02R.1289.07 Sample:TCGA.BP.4763.01A.01R.1289.07 Sample:TCGA.BP.4765.01A.01R.1289.07 Sample:TCGA.BP.4766.01A.01R.1289.07 Sample:TCGA.BP.4768.01A.01R.1289.07 Sample:TCGA.BP.4769.01A.01R.1289.07 Sample:TCGA.BP.4770.01A.01R.1503.07 Sample:TCGA.BP.4771.01A.01R.1289.07 Sample:TCGA.BP.4774.01A.01R.1289.07 Sample:TCGA.BP.4775.01A.01R.1289.07 Sample:TCGA.BP.4777.01A.01R.1289.07 Sample:TCGA.BP.4781.01A.01R.1305.07 Sample:TCGA.BP.4782.01A.02R.1420.07 Sample:TCGA.BP.4784.01A.01R.1305.07 Sample:TCGA.BP.4787.01A.01R.1305.07 Sample:TCGA.BP.4789.01A.01R.1305.07 Sample:TCGA.BP.4790.01A.01R.1305.07 Sample:TCGA.BP.4795.01A.02R.1420.07 Sample:TCGA.BP.4797.01A.01R.1305.07 Sample:TCGA.BP.4798.01A.01R.1305.07 Sample:TCGA.BP.4799.01A.01R.1305.07 Sample:TCGA.BP.4801.01A.02R.1420.07 Sample:TCGA.BP.4803.01A.01R.1305.07 Sample:TCGA.BP.4804.01A.02R.1305.07 Sample:TCGA.BP.4807.01A.01R.1305.07 Sample:TCGA.BP.4959.01A.01R.1334.07 Sample:TCGA.BP.4960.01A.01R.1334.07 Sample:TCGA.BP.4961.01A.01R.1334.07 Sample:TCGA.BP.4962.01A.01R.1334.07 Sample:TCGA.BP.4963.01A.01R.1334.07 Sample:TCGA.BP.4964.01A.01R.1334.07 Sample:TCGA.BP.4965.01A.01R.1334.07 Sample:TCGA.BP.4967.01A.01R.1334.07 Sample:TCGA.BP.4968.01A.01R.1334.07 Sample:TCGA.BP.4969.01A.01R.1334.07 Sample:TCGA.BP.4970.01A.01R.1334.07 Sample:TCGA.BP.4971.01A.01R.1334.07 Sample:TCGA.BP.4972.01A.01R.1334.07 Sample:TCGA.BP.4973.01A.01R.1334.07 Sample:TCGA.BP.4974.01A.01R.1334.07 Sample:TCGA.BP.4975.01A.01R.1334.07 Sample:TCGA.BP.4976.01A.01R.1334.07 Sample:TCGA.BP.4977.01A.01R.1334.07 Sample:TCGA.BP.4981.01A.01R.1334.07 Sample:TCGA.BP.4982.01A.01R.1334.07 Sample:TCGA.BP.4983.01A.01R.1334.07 Sample:TCGA.BP.4985.01A.01R.1334.07 Sample:TCGA.BP.4986.01A.01R.1334.07 Sample:TCGA.BP.4987.01A.01R.1334.07 Sample:TCGA.BP.4988.01A.01R.1334.07 Sample:TCGA.BP.4989.01A.01R.1334.07 Sample:TCGA.BP.4991.01A.01R.1334.07 Sample:TCGA.BP.4992.01A.01R.1334.07 Sample:TCGA.BP.4993.01A.02R.1420.07 Sample:TCGA.BP.4994.01A.01R.1334.07 Sample:TCGA.BP.4995.01A.01R.1334.07 Sample:TCGA.BP.4998.01A.01R.1334.07 Sample:TCGA.BP.4999.01A.01R.1334.07 Sample:TCGA.BP.5000.01A.01R.1334.07 Sample:TCGA.BP.5001.01A.01R.1334.07 Sample:TCGA.BP.5004.01A.01R.1334.07 Sample:TCGA.BP.5006.01A.01R.1334.07 Sample:TCGA.BP.5007.01A.01R.1334.07 Sample:TCGA.BP.5008.01A.01R.1334.07 Sample:TCGA.BP.5009.01A.01R.1334.07 Sample:TCGA.BP.5010.01A.02R.1420.07 Sample:TCGA.BP.5168.01A.01R.1420.07 Sample:TCGA.BP.5169.01A.01R.1426.07 Sample:TCGA.BP.5170.01A.01R.1426.07 Sample:TCGA.BP.5173.01A.01R.1426.07 Sample:TCGA.BP.5174.01A.01R.1426.07 Sample:TCGA.BP.5175.01A.01R.1426.07 Sample:TCGA.BP.5176.01A.01R.1426.07 Sample:TCGA.BP.5177.01A.01R.1426.07 Sample:TCGA.BP.5178.01A.01R.1426.07 Sample:TCGA.BP.5180.01A.01R.1426.07 Sample:TCGA.BP.5181.01A.01R.1426.07 Sample:TCGA.BP.5182.01A.01R.1426.07 Sample:TCGA.BP.5183.01A.01R.1426.07 Sample:TCGA.BP.5187.01A.01R.1426.07 Sample:TCGA.BP.5189.01A.02R.1426.07 Sample:TCGA.BP.5190.01A.01R.1426.07 Sample:TCGA.BP.5191.01A.01R.1426.07 Sample:TCGA.BP.5192.01A.01R.1426.07 Sample:TCGA.BP.5194.01A.02R.1426.07 Sample:TCGA.BP.5195.01A.02R.1426.07 Sample:TCGA.BP.5196.01A.01R.1426.07 Sample:TCGA.BP.5198.01A.01R.1426.07 Sample:TCGA.BP.5199.01A.01R.1426.07 Sample:TCGA.BP.5200.01A.01R.1426.07 Sample:TCGA.BP.5201.01A.01R.1426.07 Sample:TCGA.BP.5202.01A.02R.1426.07 Sample:TCGA.CJ.4634.01A.02R.1325.07 Sample:TCGA.CJ.4635.01A.02R.1305.07 Sample:TCGA.CJ.4636.01A.02R.1325.07 Sample:TCGA.CJ.4637.01A.02R.1325.07 Sample:TCGA.CJ.4638.01A.02R.1325.07 Sample:TCGA.CJ.4639.01A.02R.1325.07 Sample:TCGA.CJ.4640.01A.02R.1325.07 Sample:TCGA.CJ.4641.01A.02R.1325.07 Sample:TCGA.CJ.4643.01A.02R.1325.07 Sample:TCGA.CJ.4644.01A.02R.1325.07 Sample:TCGA.CJ.4868.01A.01R.1305.07 Sample:TCGA.CJ.4869.01A.02R.1426.07 Sample:TCGA.CJ.4871.01A.01R.1305.07 Sample:TCGA.CJ.4872.01A.01R.1305.07 Sample:TCGA.CJ.4873.01A.01R.1305.07 Sample:TCGA.CJ.4874.01A.01R.1305.07 Sample:TCGA.CJ.4875.01A.01R.1305.07 Sample:TCGA.CJ.4876.01A.01R.1305.07 Sample:TCGA.CJ.4878.01A.01R.1305.07 Sample:TCGA.CJ.4881.01A.01R.1305.07 Sample:TCGA.CJ.4882.01A.02R.1426.07 Sample:TCGA.CJ.4884.01A.01R.1305.07 Sample:TCGA.CJ.4885.01A.01R.1305.07 Sample:TCGA.CJ.4886.01A.01R.1305.07 Sample:TCGA.CJ.4887.01A.01R.1305.07 Sample:TCGA.CJ.4888.01A.01R.1305.07 Sample:TCGA.CJ.4889.01A.01R.1305.07 Sample:TCGA.CJ.4890.01A.01R.1305.07 Sample:TCGA.CJ.4891.01A.01R.1305.07 Sample:TCGA.CJ.4892.01A.01R.1305.07 Sample:TCGA.CJ.4893.01A.01R.1305.07 Sample:TCGA.CJ.4894.01A.01R.1305.07 Sample:TCGA.CJ.4895.01A.01R.1305.07 Sample:TCGA.CJ.4897.01A.03R.1426.07 Sample:TCGA.CJ.4899.01A.01R.1334.07 Sample:TCGA.CJ.4900.01A.01R.1334.07 Sample:TCGA.CJ.4901.01A.01R.1426.07 Sample:TCGA.CJ.4902.01A.01R.1426.07 Sample:TCGA.CJ.4903.01A.01R.1426.07 Sample:TCGA.CJ.4904.01A.02R.1426.07 Sample:TCGA.CJ.4905.01A.02R.1426.07 Sample:TCGA.CJ.4907.01A.01R.1426.07 Sample:TCGA.CJ.4908.01A.01R.1426.07 Sample:TCGA.CJ.4912.01A.01R.1426.07 Sample:TCGA.CJ.4916.01A.01R.1426.07 Sample:TCGA.CJ.4918.01A.01R.1426.07 Sample:TCGA.CJ.4920.01A.01R.1426.07 Sample:TCGA.CJ.4923.01A.01R.1426.07 Sample:TCGA.CJ.5671.01A.11R.1541.07 Sample:TCGA.CJ.5672.01A.11R.1541.07 Sample:TCGA.CJ.5675.01A.11R.1541.07 Sample:TCGA.CJ.5676.01A.11R.1541.07 Sample:TCGA.CJ.5677.01A.11R.1541.07 Sample:TCGA.CJ.5678.01A.11R.1541.07 Sample:TCGA.CJ.5679.01A.11R.1541.07 Sample:TCGA.CJ.5680.01A.11R.1541.07 Sample:TCGA.CJ.5681.01A.11R.1541.07 Sample:TCGA.CJ.5682.01A.11R.1541.07 Sample:TCGA.CJ.5683.01A.11R.1541.07 Sample:TCGA.CJ.5684.01A.11R.1541.07 Sample:TCGA.CJ.5686.01A.11R.1672.07 Sample:TCGA.CJ.5689.01A.11R.1541.07 Sample:TCGA.CJ.6027.01A.11R.1672.07 Sample:TCGA.CJ.6028.01A.11R.1672.07 Sample:TCGA.CJ.6030.01A.11R.1672.07 Sample:TCGA.CJ.6031.01A.11R.1672.07 Sample:TCGA.CJ.6032.01A.11R.1672.07 Sample:TCGA.CJ.6033.01A.11R.1672.07 Sample:TCGA.CW.5580.01A.01R.1672.07 Sample:TCGA.CW.5581.01A.02R.1541.07 Sample:TCGA.CW.5583.01A.02R.1541.07 Sample:TCGA.CW.5584.01A.01R.1541.07 Sample:TCGA.CW.5585.01A.01R.1541.07 Sample:TCGA.CW.5587.01A.01R.1541.07 Sample:TCGA.CW.5589.01A.01R.1541.07 Sample:TCGA.CW.5590.01A.01R.1541.07 Sample:TCGA.CW.5591.01A.01R.1541.07 Sample:TCGA.CW.6087.01A.11R.1672.07 Sample:TCGA.CW.6088.01A.11R.1672.07 Sample:TCGA.CW.6090.01A.11R.1672.07 Sample:TCGA.CW.6093.01A.11R.1672.07 Sample:TCGA.CW.6097.01A.11R.1672.07 Sample:TCGA.CZ.4853.01A.01R.1426.07 Sample:TCGA.CZ.4854.01A.01R.1305.07 Sample:TCGA.CZ.4856.01A.02R.1426.07 Sample:TCGA.CZ.4857.01A.01R.1305.07 Sample:TCGA.CZ.4858.01A.01R.1305.07 Sample:TCGA.CZ.4859.01A.02R.1426.07 Sample:TCGA.CZ.4860.01A.01R.1305.07 Sample:TCGA.CZ.4861.01A.01R.1305.07 Sample:TCGA.CZ.4862.01A.01R.1305.07 Sample:TCGA.CZ.4863.01A.01R.1503.07 Sample:TCGA.CZ.4864.01A.01R.1503.07 Sample:TCGA.CZ.4865.01A.02R.1503.07 Sample:TCGA.CZ.4866.01A.01R.1503.07 Sample:TCGA.CZ.5452.01A.01R.1503.07 Sample:TCGA.CZ.5453.01A.01R.1503.07 Sample:TCGA.CZ.5454.01A.01R.1503.07 Sample:TCGA.CZ.5455.01A.01R.1503.07 Sample:TCGA.CZ.5457.01A.01R.1503.07 Sample:TCGA.CZ.5458.01A.01R.1503.07 Sample:TCGA.CZ.5460.01A.01R.1503.07 Sample:TCGA.CZ.5461.01A.01R.1503.07 Sample:TCGA.CZ.5462.01A.01R.1503.07 Sample:TCGA.CZ.5463.01A.01R.1503.07 Sample:TCGA.CZ.5464.01A.01R.1503.07 Sample:TCGA.CZ.5465.01A.01R.1503.07 Sample:TCGA.CZ.5467.01A.01R.1503.07 Sample:TCGA.CZ.5468.01A.01R.1503.07 Sample:TCGA.CZ.5469.01A.01R.1503.07 Sample:TCGA.CZ.5470.01A.01R.1503.07 Sample:TCGA.CZ.5982.01A.11R.1672.07 Sample:TCGA.CZ.5984.01A.11R.1672.07 Sample:TCGA.CZ.5985.01A.11R.1672.07 Sample:TCGA.CZ.5986.01A.11R.1672.07 Sample:TCGA.CZ.5987.01A.11R.1672.07 Sample:TCGA.CZ.5988.01A.11R.1672.07 Sample:TCGA.CZ.5989.01A.11R.1672.07 Sample:TCGA.DV.5565.01A.01R.1541.07 Sample:TCGA.DV.5566.01A.01R.1541.07 Sample:TCGA.DV.5567.01A.01R.1541.07 Sample:TCGA.DV.5568.01A.01R.1541.07 Sample:TCGA.DV.5569.01A.01R.1541.07 Sample:TCGA.DV.5573.01A.01R.1541.07 Sample:TCGA.DV.5574.01A.01R.1541.07 Sample:TCGA.DV.5575.01A.01R.1541.07 Sample:TCGA.DV.5576.01A.01R.1541.07 FILTER:mean FILTER:sd FILTER:cv FILTER:unif.mean FILTER:unif.sd FILTER:unif.cv FILTER:q5freq FILTER:q4freq FILTER:q3freq FILTER:q2freq FILTER:RT.PCR.OK FILTER:RT.PCR.OK.2 FILTER:RT.PCR.OK.4 FILTER:RT.PCR.OK.5 FILTER:ftest.GRADE FILTER:ftest.PATHOLOGY FILTER:ftest.STAGE FILTER:ftest.EVENT FILTER:kruskal.GRADE FILTER:kruskal.PATHOLOGY FILTER:kruskal.STAGE FILTER:kruskal.EVENT FILTER:spearman.AGE FILTER:pearson.AGE FILTER:disparse.level FILTER:cox.SURVIVAL_MONTHS FILTER:cox5.SURVIVAL_MONTHS FILTER:Corr0.5 FILTER:Corr0.7 FILTER:Corr0.9
